# Materials Used in Electric Vehicle Battery Housings: Recycling Pathways and Circular Design—A Review

**DOI:** 10.3390/ma19132808

**Published:** 2026-07-02

**Authors:** Patrycja Bazan, Agnieszka Przybek, Michał Łach, Kamil Badura, Piotr Duda, Piotr Bielaczyc

**Affiliations:** 1 Department of Materials Engineering, Faculty of Materials Science and Physics, Cracow University of Technology, al. Jana Pawła II 37, 31-864 Kraków, Poland; patrycja.bazan@pk.edu.pl (P.B.); agnieszka.przybek@pk.edu.pl (A.P.); kamil.badura@doktorant.pk.edu.pl (K.B.); 2 Interdisciplinary Center for Circular Economy, Cracow University of Technology, ul. Warszawska 24, 31-155 Kraków, Poland; 3 CUT Doctoral School, Cracow University of Technology, Warszawska 24, 31-155 Cracow, Poland; 4 Chair of Thermal and Process Engineering, Faculty of Mechanical Engineering, Cracow University of Technology, al. Jana Pawła II 37, 31-864 Cracow, Poland; piotr.duda@pk.edu.pl; 5 BOSMAL Automotive Research & Development Institute Ltd., 43-300 Bielsko-Biala, Poland; 6 Department of Motor Vehicles, Faculty of Mechanical Engineering, Cracow University of Technology, al. Jana Pawła II 37, 31-864 Cracow, Poland

**Keywords:** electric vehicle battery housing, circular economy, recycling pathways, sustainable materials, design for recycling

## Abstract

Battery housings are critical structural and safety components in electric vehicles, fulfilling multiple functions related to mechanical protection, crashworthiness, thermal management, fire resistance, electromagnetic shielding, and integration of battery modules into the vehicle body. While metallic housings, particularly aluminum and steel, remain dominant in industrial applications, increasing attention is being given to composite materials as lightweight alternatives capable of improving energy efficiency and extending driving range. However, the growing use of composites in battery enclosures raises important questions regarding recyclability, end-of-life management, and compatibility with circular economy principles. This review critically examines the current state of the art in composite materials used for electric vehicle battery housings, with particular emphasis on glass- and carbon-fiber-reinforced thermoplastics, thermoset composites, sandwich structures, and hybrid multi-material systems. The paper discusses the functional requirements imposed on battery housings and analyzes how these requirements influence material selection and design strategies. Particular attention is devoted to recycling pathways applicable to composite battery enclosures, including mechanical recycling, thermal treatment, chemical recycling, and reuse-oriented approaches, as well as to the limitations associated with mixed-material assemblies, adhesives, coatings, and integrated functions. The review also addresses circular design strategies for battery housings, including design for disassembly, material traceability, modularity, and the incorporation of recycled polymers and secondary reinforcements into new housing systems. Current research gaps are identified in the integration of structural performance, fire safety, manufacturability, and recyclability within a single design framework. The analysis shows that thermoplastic composites currently offer the most promising route toward circular battery enclosures, while thermoset-based systems still face significant challenges in high-value recycling. The paper concludes by outlining future research directions required for the development of lightweight, safe and recyclable composite battery housings aligned with sustainable mobility and circular economy goals.

## 1. Introduction

The rapid electrification of transportation poses significant challenges for energy storage systems, particularly concerning vehicle components such as battery pack housings. The imperative for high energy density and structural rigidity, coupled with the necessity to reduce weight, is driven by regulatory, economic, and environmental pressures, thereby propelling the automotive industry towards the adoption of lightweight construction and multifunctional materials [1]. The anticipated global battery demand, projected to exceed 1 TWh by mid-decade, further necessitates considerations of safety, thermal management and durability [2]. Traditional metal enclosures are constrained by mass and functional limitations. In contrast, composite materials, characterized by high strength-to-weight ratios, are emerging as innovative solutions, fulfilling structural, protective and environmental roles. Research into lightweight composites for electromobility has extensively examined carbon, glass and natural fiber-reinforced composites with resins for application in battery housings and vehicle components. Emphasis is placed on integrating thermal stability, electromagnetic shielding and recyclability within a single material [3]. The reduction of battery enclosure weight is critical in electric vehicles, where the battery pack can constitute up to 30% of the total weight, thereby heightening interest in lightweight structures [1]. Concurrently, analyses of policies and supply chains underscore the critical importance of raw materials (lithium, nickel, cobalt) and the necessity for material solutions that promote a circular economy, including the facilitation of dismantling and recycling of package components, such as battery packaging [2]. From this perspective, fiber composite enclosures must be evaluated not solely through the lens of weight reduction but as part of a comprehensive design strategy aimed at reuse, recycling and minimizing environmental impact. The concept of sustainable materials for battery structures also encompasses the transition towards bio-based resins and reinforcements and the advancement of composite recycling technology [4]. Proposed developmental directions include the intensification of recovery methods, enhancement of component compatibility for easier disassembly and the design of hybrid composite systems that balance strength requirements with recyclability [5]. Economic analyses and circular economy scenarios indicate that repurposing used batteries from electric vehicles for energy storage in photovoltaic installations could capture a significant portion of surplus energy production. In the proposed simulations, this proportion ranges from a few to over twenty percent, suggesting a viable approach to reducing the demand for new batteries [6]. This solution could alleviate the pressure on raw material extraction and decrease the volume of battery waste treatment. However, its implementation necessitates extensive logistical infrastructure, regulatory frameworks, and economic instruments to facilitate the safe and efficient transfer of components between the automotive and energy sectors. Coupled with legal and market requirements for closing the material loop in the battery sector, this context shapes the framework for research into battery enclosures as critical elements of the circular economy [2].

Due to the interdisciplinary nature of electric vehicle battery housing design, a number of technical abbreviations are used throughout this review. To improve readability and facilitate navigation through the manuscript, a summary of the abbreviations and their corresponding definitions is provided in Table 1.

## 2. The Growing Role of Battery Housing in Electric Vehicles

### 2.1. The Importance of Housing in the Context of Safety

The safe operation of battery systems requires the housing to act as the final barrier against ignition or explosion. Vibroacoustic effects from road irregularities and the drive system cause deformation and stress, necessitating a design that prevents damage to the package’s integrity. A robust design protects cells from impacts and punctures, preventing damage and ignition. The housing minimizes heat loss, maintaining safe temperatures and preventing thermal runaway. It insulates against short circuits, moisture, and contaminants that could cause corrosion or performance issues. Modern designs include sensors to monitor battery status, enabling rapid protective actions and providing space for swelling or gas release, enhancing safety [7]. A typical package features a top enclosure, protecting components from impacts, dust, and moisture, serving as an electromagnetic shield and a mounting surface for sensors. The frame and bottom plate provide rigidity, crash protection, and vibration resistance, with the bottom able to accommodate fire-retardant layers. Cooling systems beneath or between modules, using liquid cooling plates or refrigerant channels, are crucial for maintaining safe temperatures. This highlights the importance of thermal layers and mounting solutions for constant contact between cooling plates and cells [8]. The enclosure’s significance in emergencies is evident in fire incidents involving vehicles. A high-speed collision can breach a battery, causing gas escape and thermal runaway, with repeated ignition during towing due to residual energy and unclear high-voltage cut-off markings complicating isolation. This underscores the need for clear high-voltage insulation design, marked cut-off points, automatic disconnection post-collision, and effective short-circuit detection to protect users and rescuers [9]. The battery management system’s (BMS’s) security is linked to the enclosure, as it monitors voltages, currents, and temperatures to prevent overheating and execute protective actions [9,10]. Reliable hardware and software interfaces are critical for safety. Therefore, package security requires integrating the enclosure, BMS, and thermal system as a unified protection system [2,8,9,10].

### 2.2. The Impact of Enclosures on Vehicle Performance and Efficiency

The weight and design of the battery housing are crucial for electric vehicle performance, comprising 25–35% of total weight. Enhancing gravimetric energy density and range or reducing package size and cost while maintaining range is essential [2,11]. Reducing weight by one kilogram can cut costs, promoting lightweight enclosure solutions [11]. Composite materials like carbon fiber-reinforced polymers (CFRPs) are viable alternatives to metals [1]. Despite higher energy production needs, replacing steel with these materials can lower life-cycle emissions by reducing operating energy. Lightweight geometries also minimize structural material needs and drive size. Enclosures impact more than only weight reduction. Innovations in composite housings and graphene laminates enhance system performance without altering cell chemistry energy density. Composites with superior barrier properties and stability enable compact packaging, benefiting energy storage. Lightweight, multifunctional enclosures reduce vehicle weight and emissions. Efficient design and materials can significantly cut emissions, even with higher production energy [12]. In electric vehicles, lightweight materials boost autonomy by reducing weight and decreasing battery pack weight with the same chemistry improves performance alongside electrode composition changes [13]. However, new cell technologies, like solid-state concepts, involve energy-intensive processes such as high-temperature sintering, affecting mass reduction benefits [14]. Enclosure designs must consider production energy use to avoid increased environmental footprints. Battery production is energy and emission-intensive, with estimates of 350–650 MJ per kWh and 150–200 kg of CO_2_ per kWh, resulting in several tons of CO_2_ emissions for smaller EV batteries [15]. Thus, material and process choices for enclosures should be life-cycle oriented, as mass reduction benefits may be offset by larger production footprints. Literature analyses, including SWOT analysis and material benchmarking, show that design decisions must balance weight reduction with environmental and cost impacts [16].

### 2.3. Evolution of Housing Design

With the increasing number of electric vehicles, package housing design is also evolving. Analyses show that module enclosures, thermal systems, cells, and BMS are distinct, moving from a simple “box” to a complex structure [17]. Design trends reveal that enclosures now include mechanical safety, cooling, and collision load paths, beyond cell protection [18,19]. Nowadays, advanced methodologies use multi-stage numerical analysis, modal analysis, frequency response per UN ECE R100, and crash simulations. Modal analysis assesses housing rigidity and weight to avoid natural vibration frequencies coinciding with road exertion; they should exceed 7 Hz, with torsional modes outside 20–40 Hz [20]. This optimizes enclosures for dynamic and regulatory needs. Enclosures are also vital in heat management and thermal safety. Reviews note thermal barriers like low-conductivity layers or PCMs mitigate thermal runaway, lowering cell temperatures [21]. Composite and metal cooling systems with PCM and metallic foam may enhance thermal conductivity. For example, PCM composites with fillers like graphene offer better conductivity and stability, suggesting integration into battery housing. Modules filled with phase change material create heat storage units with 120–220 kWh/m energy density, outperforming traditional solutions in district heating. Regarding package reuse concepts use existing metal enclosures for new applications. Research on recycling traction modules for heat storage examines steel or aluminum housings initially for cells and cooling [22]. This extends the enclosure’s life cycle, which is essential for thermal energy storage post-traction. As presented, the material and environmental demands for enclosure construction are changing. Reviews on Lightweight Materials for E-Mobility discuss mainly CFRP, GFRP, and hybrid composites in battery compartments for insulation, fire resistance, and shielding. These studies stress the importance of life cycle assessment, recycling, and bio-based systems, affecting future enclosure designs [1,4]. Thus, enclosure design progresses from a rigid box to a multi-material module, tailored for dynamics, safety, thermal management, and further use [4,17,20,22].

### 2.4. Numerical Modeling of Battery Casings

Much of the research on lithium-ion batteries focuses on the analysis of electrochemical phenomena, although their failure and thermal runaway are often caused by mechanical loading. A review of mechanical modeling of these batteries at different length scales is presented in [23]. Battery housings must be able to withstand significant weight and maintain their structural integrity. The deformation process of the battery pack structure is simulated using a Finite Element Method (FEM) [24]. Experimentally verified simulations show that the blast-resistant adaptive sandwich prevents buckling and ensures a stable load-bearing capacity, which translates into high impact resistance. In [25], the use of carbon fiber for the production of battery casings is proposed. The first part of this paper presents the results of thermoforming simulations by PAM-FORM, while the second part is devoted to crashworthiness modeling by Structural Mechanics (VPS). Numerical tests are performed for a side impact with a pole at impact speeds ranging from 15 to 35 mph. Combining two FEM models—one for thermoforming processes and one for impact—made it possible to determine the effect of material structure on impact resistance. The study shows that the specific absorbed energy is inversely proportional to the number of layers in the carbon fiber composite. Numerical modeling is also used for composite-battery integrated structures [26]. Batteries are modeled as a homogenized material using foam-based FEM models with LS-Dyna and ABAQUS. The simulation accurately predicts the behavior during an impact, and the maximum error in the peak impact load does not exceed 8%. Article [27] presents a strength analysis of a battery casing made of a hybrid thermoplastic composite, designed for transportation applications. Various load scenarios are considered, including sinusoidal and random vibrations, mechanical shocks, and impact loads. FEM analyses performed using ANSYS software have shown that this thermoplastic battery enclosure is suitable for transportation applications. Impact analysis of a battery casing made of composite material using Sheet Molding Technology (SMC) is shown in [28]. Drop-tower impact tests are conducted on flat SMC composite plates and the housing, thereby validating the numerical models. Parametric modeling of ground impacts revealed a mechanism of gradual damage at low velocities, until the point where internal damage becomes likely at the critical velocity. The paper [29] presents a topological optimization of a lightweight battery pack enclosure made of carbon-fiber-reinforced plastic. Based on specific boundary conditions and load cases, an isotropic material model is applied using the penalty method. The FEM model in Abaqus is used to verify stiffness and strength, perform buckling analysis, and analyze bolt loads, thereby ensuring the reliability of the connections. Experimental tests on the developed design confirm its strength. Another effective example of the use of numerical methods to reduce weight and conduct strength tests on a battery-pack system is the study in [30]. The calculations are performed using the FEM method with LSDYNA software. The structural optimization algorithm is based on two methods described in the cited literature: orthogonal experimental design and response surface methodology. The prototype is manufactured, but its correctness is verified in this study only numerically. In [31], machine learning is successfully applied to the production of battery casings made of carbon fiber composite. The algorithm uses the results of previous FEM analyses to simulate thermoforming and impact. Publication [32] presents a design for a battery box for an electric vehicle made of glass-fiber-reinforced polymer. A total of 27 models are used for multi-criteria optimization using artificial neural networks, resulting in the development of an optimized design. Thanks to this design, the side profile of the battery box becomes 23.9% lighter and 38.6% cheaper, while its performance increases by 3%. Some studies have highlighted the need to model the Life Cycle Analysis (LCA) of the enclosures used. The LCA of a battery casing manufactured from composite materials using the pultrusion method, involving an estimation of CO_2_ emissions and cumulative energy demand, is presented in [33]. The battery pack design is developed using SOLIDWORKS, and the LCA is conducted using the EuCIA’s Eco Impact Calculator. Multifunctional carbon fiber composites can reduce the environmental impact over their entire life cycle and lower energy consumption compared to traditional materials [34]. Apart from studies that numerically model plastic battery casings or conduct an LCA, there are currently no publications presenting the results of modeling casings made from recycled materials. Further research should focus on developing numerical models for actual mixtures of recycled materials and verifying them experimentally. Numerical modeling of plastic battery enclosures, including those made from recycled materials, plays a key role in the development of safe, lightweight, and environmentally friendly solutions for electric vehicles.

As shown in Table 2, no single modeling approach is capable of addressing all design requirements associated with EV battery housings. Finite Element Method (FEM) simulations remain the dominant tool for structural integrity assessment, crashworthiness analysis, vibration behavior, and thermo-mechanical performance evaluation due to their high predictive accuracy and strong validation against experimental data. However, these methods are often computationally intensive, particularly when multi-physics phenomena are considered. In contrast, machine learning and surrogate modeling approaches offer significantly reduced computational times and are increasingly used during early-stage design optimization and rapid parameter screening. Nevertheless, their predictive capabilities strongly depend on the availability and quality of training datasets. Therefore, current research trends increasingly favor hybrid frameworks combining physics-based FEM models with machine-learning-assisted optimization techniques, enabling both high accuracy and computational efficiency in the development of next-generation battery enclosure systems.

## 3. Functional Complexity of Battery Enclosure Systems

### 3.1. Mechanical Components

**Figure 1:**
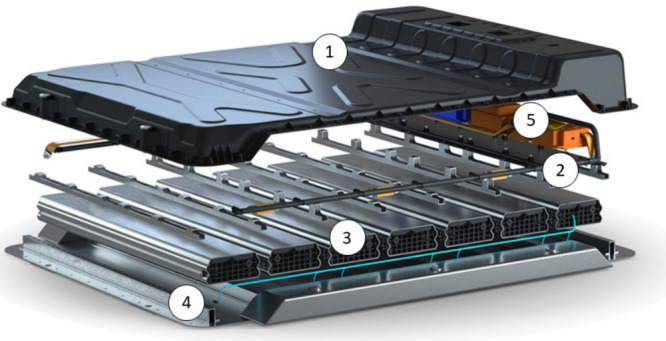
Overview of a typical lithium-ion battery pack housing [2,7,17,37] (schematic illustration intended to present the functional architecture of a typical EV battery pack housing).

The typical architecture of a traction battery package includes cell modules, BMS, cooling, and an enclosure that provides mechanical protection, tightness, and secure installation in the vehicle is shown in Figure 1, while Table 3 presents functional requirements for EV battery enclosures.

Key components to visualize:

1. Top cover—Protection against external factors, EMI shielding, sensor mounting.
2. Frame/Load-bearing structure—Load-bearing capacity, side impact protection.
3. Cell Modules and BTMS—Integration of cells into the cooling plate (fluid/PCM) for thermal stabilization.
4. Bottom plate with cover—Ballistic protection, thermal runaway mitigation.
5. BDU (Battery Disconnected Unit)—HV Connectors, Fuses and Power Management Systems.

**Table 3:** Functional requirements for EV battery enclosures.

Category	Parameters/Description	Standards/Tests	Implications of Materials	Literature
Mechanical	Stiffness, fatigue, vibration (>7 Hz, 20–40 Hz torsional modes)	UN ECE R100	High specific stiffness	[7]
Crash	Protection at side pole impact	FMVSS 214, EuroNCAP	Crumple zones, energy absorption (SEA)	[38]
Thermal	Operating range 10–40 °C; temperature gradient < 5 °C	-	Thermal conductivity, thermal insulation	[21]
Fire	Thermal runaway containment	UN GTR 20	Barrier layers, PCM, fire resistance	[37]
Environmental	Corrosion resistance, road salt and moisture resistance	ISO 16750	Composites, advanced protective coatings	[1]
Electric	High Voltage (HV) Isolation, EMI Shielding	ISO 11452	Conductive layers, grounding, rGO	[10]
Production	Cycle time, tooling cost, seriality	-	RTM, SMC, pultrusion methods	[6]

The battery housing must transfer loads, protect cells, and remain lightweight. In electromobility, it must endure vibrations, impacts, temperature changes, and environmental factors without adding significant weight. That is why fiber composites are crucial here. Carbon Fiber-Reinforced Polymer (CFRP) and Glass Fiber-Reinforced Polymer (GFRP) offer high strength-to-weight ratios, fatigue and corrosion resistance, and anisotropy-enhancing load paths [39]. Biocomposites, though weaker, are also used, but in less critical parts, for their low environmental impact and vibration damping [4,40]. Material and fiber selection balances mechanical properties, weight, cost, and sustainability. Anisotropy affects stiffness in key load directions. Stress trajectory fibers increase local stiffness with minimal material, vital for battery components. An improved damping reduces vibration, extending housing and battery life [3,4,39]. Hybrid systems combine composites with aluminum or steel, utilizing metal stiffness and ductility in collision areas and composite lightness elsewhere. Finite element method (FEM) simulations show that hybrids surpass all-metal enclosures in fatigue strength and crash resistance while being lighter, which is ideal for battery support structures [39]. Integrating functions like module support and collision force transmission requires careful composite-metal connection design to avoid stress concentration and delamination. FEM and topology optimization identify load paths, removing low-stress material, optimizing weight, vibration frequency, and displacement for a light, rigid structure meeting safety and comfort needs [35]. Recent developments in topology optimization have additionally incorporated adaptive stress constraints and manufacturing limitations, allowing designers to generate lightweight structures that remain feasible for industrial production. Such approaches have been successfully demonstrated for highly loaded transport components and may provide a useful framework for the optimization of EV battery housings, where structural efficiency, crashworthiness and sustainability objectives must be simultaneously considered [41,42]. Research on lightweight materials, like additively manufactured foam and truss structures, suggests potential for future enclosures, reducing weight while maintaining stiffness-to-weight ratios and energy absorption, crucial in collision and support zones [11].

Essential function serves protection components. The crash protection of battery packs depends on components designed to manage deformation and absorb energy. Pole side impact tests assess vehicles, confirming the enclosure’s capacity for energy absorption, deformation reduction, and structural integrity maintenance. Batteries beneath the floor aim to preserve cell integrity while allowing housing deformation to distribute energy absorption among elements like the floor, roof, or body pillars [20,38]. Design strategies include reinforcing the structure and incorporating “sacrificial” elements. Filling thin-walled profiles with metallic foam or honeycomb in door thresholds and using thin-walled pipe contours enhances compression resistance and protection. Research suggests adding new energy-absorbing elements in the floor and thresholds and using tapered corrugated tubes to mitigate accelerations in pole side impact tests [32,38]. Other studies emphasize protective elements by examining materials and thicknesses of enclosure sheets for protection. Optimizing package module shapes for lateral impact shows that hexagonal geometry offers superior energy absorption and space utilization, highlighting geometric architecture’s importance in impact protection [18]. Designing protective frames as crumple zones involves determining strain space and allocating it between module mounting and energy-absorbing zones. While frame construction analyses show that closed, thin-walled profiles (e.g., pipes) deform without losing continuity, facilitating stable energy absorption. Multi-chamber cross-sections enhance gradual deformation potential but require careful cross-sectional area and material selection to prevent brittle cracking under impact [20]. Fiberglass-reinforced composites are promising for battery side protection. The GFRP side profile design and optimization show these elements enhance housing durability while reducing weight, improving vehicle energy efficiency. Pultrusion technology enables long, continuous profiles with homogeneous microstructure, high strength, and corrosion resistance, serving as lightweight, load-bearing protective elements in side zones. Research on thin-walled pipes with trusses or auxetic structures shows significant energy absorption increases over hollow profiles. Studies on pipe crushing behavior indicate that internal cages enhance crash performance, relevant for reinforcing profiles around battery housings. Topological optimization methods for collision elements use discrete algorithms to maximize energy absorption within mass and strain constraints [32]. Housing material choice also affects thermal conductivity and impact resistance; aluminum enclosures or foam sandwich panels improve heat dissipation and crush resistance, beneficial for floor-mounted faucets [43]. However, these solutions may involve weight, cost trade-offs, and chassis integration considerations. Reviews suggest effective package protection arises from interaction between housing and vehicle chassis, including thresholds and floor crossbars. An architectural approach is recommended, integrating energy-absorbing elements into both enclosures and supporting profiles, with joint optimization of geometric and material parameters for crash scenarios, like side impacts with poles [38].

### 3.2. Electrical Components

**Figure 2:**
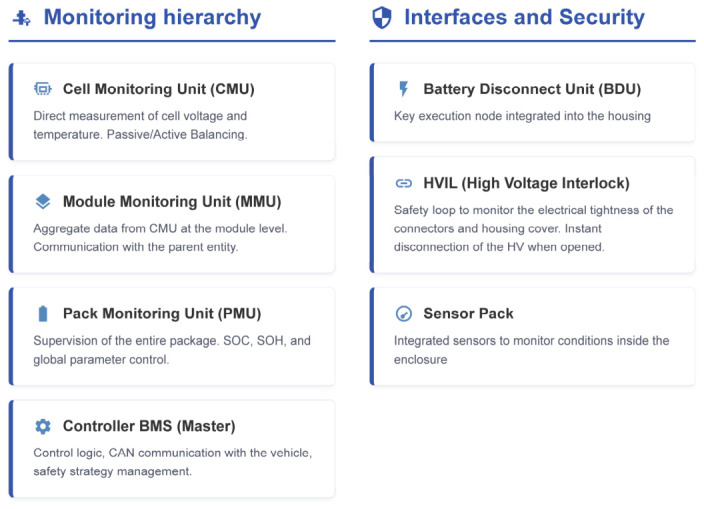
Integration of the BMS system into the housing [9,10,44,45,46].

Within the traction package, the Battery Management System (BMS) is a crucial interface between the measurement and diagnostic layer and the actuators, ensuring safe battery integration with the high-voltage system. It includes hardware and software to monitor battery safety, manage cell performance for longevity, enhance capacity, and ensure safe operation [35]. Key BMS functions are voltage and current measurement, state of charge determination, and temperature monitoring, requiring precise signals like package current and cell voltages [10]. Sensor data on voltage, temperature, current, and humidity are processed in the data acquisition layer for the battery management controller’s decisions [44]. The BMS dictates electrical and control component integration within the package. The package structure accommodates specialized electrical components with appropriate housing solutions. Key components include contactors for disconnection, fuses for overcurrent protection, a pre-charging system, current sensors, an isolation monitor, and a battery management controller [44,45]. The high-voltage distribution system (BDU) includes contactors, fuses, connectors, disconnectors, and HV measurement points for safe connection and disconnection to the vehicle’s HV bus, with the BDU inside or outside the package housing. The housing design must allow service access to the BDU, ensure electrical isolation, and integrate BMS circuits. A critical integration aspect is implementing high-voltage disconnection if operational limits are exceeded. Functional safety analyses emphasize isolating HV+ and HV− terminals for complete disconnection, even if a contactor is locked closed [10,35]. Using contactors with auxiliary contacts to detect the main contacts’ position and diagnose abnormal states is advisable, affecting harness routing and connector placement within the housing. A power resistor for pre-charging limits surge current on activation and safely discharges capacitance post-shutdown, crucial for maintenance procedures involving case access [10]. These considerations affect the positioning of high-voltage chambers, ventilation ducts, and insulation barriers within the mechanical framework. Besides managing energy flow, the Battery Management System (BMS) includes monitoring and protection via sensors on and within the housing. A standard setup has a low-voltage beam for cell voltage and temperature assessments via Cell Supervisory Circuit (CSC) systems, terminal current sensors, an insulation monitor, and a high-voltage Interlock (HVIL) circuit for detecting enclosure or safety cover openings [45]. The top housing serves as a mounting platform for sensors and connectors, providing electromagnetic shielding for control systems, preserving BMS measurement signal quality [2]. Integrating these functions into a single chip requires chassis design, ensuring separation of high-voltage (HV) and low-voltage (LV) paths, harness routing, service connector placement, and components for detecting openings. BMS functional safety standards, like ISO 26262 and IEC 61508, emphasize data acquisition reliability and protection function efficacy [9,46]. Electric vehicle fire case studies show that poor high-voltage insulation, inadequate insulation damage monitoring, and delays in BMS-initiated automatic HV disconnection can lead to energy retention and spontaneous ignitions, underscoring the need for a well-defined BMS interface with the HV circuit and housing [9,46]. Thus, BMS integration into the housing demands a coherent design of current paths, sensors, disconnection systems, and interlocks within a unified safety system. Analyses of module and cell connection designs show that housing design, cell arrangement, and connection solutions directly impact internal resistance, connection losses, and module disassembly and recycling; hence, BMS requirements for measurement points, diagnostic points, and cable design should be considered early in the mechanical concept of the enclosure, especially with printed circuits and high-cell solutions [47]. Optimization analyses suggest evolutionary algorithms like NSGA-II can effectively aid in package-level enclosure design, balancing heat dissipation, weight, and auto-disassembly capability for recycling [36]. Practically, digital modeling and cloud solutions for simulating aging and temperature distribution are advisable, aiding in aligning the BMS data acquisition strategy with actual design constraints. Figure 2 points to an integration of the BMS system into the housing.

Another key function in electrical components is insulation and short-circuit protection. Effective electrical insulation in high-voltage systems is crucial for battery pack safety and short-circuit protection. A high-voltage current path includes connections between the package and receiver, using conductors with larger cross-sections and components like contactors and fuses, which must meet stringent insulation standards. Functional safety analysis shows that insulation component malfunctions, such as relays and sensors, can lead to safety failures, potentially causing fire or explosion. Proper division into HV and LV circuits, isolation spacing, and diagnostics are essential for electrical design [44]. The disconnection system isolates both poles of the package, interrupting charging and discharging if limits are exceeded, using relays or contactors with high switching capacity. Functional safety analysis stresses disconnecting HV+ and HV− poles for complete separation, even if one contactor is locked. Relays with auxiliary contacts can detect main contact positions and identify faulty switches [10]. Enclosure design must ensure HV cable routing, separation from low-voltage circuits, and space for disconnect elements while maintaining spacing and insulation [44]. Housing materials and coatings, like GO/rGO on carbon fiber or PA/PE laminates, affect moisture resistance and dielectric properties, influencing insulation durability and short-circuit risk.

### 3.3. Thermal Components

In electric vehicle batteries, thermal components such as cooling plates, liquid channels, thermal interface materials (TIM), and temperature sensors play a crucial role in dissipating heat, maintaining the optimal temperature of the cells, and preventing the package from overheating. Table 4 presents a concise classification of battery thermal management systems (BTMSs).

Maintaining cell temperature within the recommended range requires various thermal management strategies, with coolant selection and package housing integration being crucial. These strategies include passive and active air cooling, liquid systems with cooling plates, phase-change material (PCM) systems, direct refrigerant cooling, and thermoelectric techniques [46]. Most lithium-ion chemistries operate optimally at 10–40 °C, needing cooling during high current loads [50]. NMC cells can reach 50–60 °C during rapid charging, requiring active cooling or housings with better thermal conductivity in compact machines. LFP cells, with high thermal stability, operate efficiently at 35–45 °C, favoring passive heat dissipation [39]. In air systems, passive cooling uses natural convection, while active cooling employs forced airflow integrated with vehicle HVAC systems. Passive solutions are simple, cost-effective, and lightweight but have low cooling efficiency at high power densities [50]. Forced airflow systems in vehicles use fans and ducts to direct air through modules, often using HVAC-air-conditioned cabin air. Research shows duct geometry, inlet/outlet angles, and cell spacing affect temperature uniformity, requiring flow system optimization during housing design [21,48]. Liquid cooling systems, using a water/glycol mixture, offer superior heat capacity and transfer efficiency compared to air, becoming common in traction applications. A typical setup includes cooling plates, supply/discharge piping, and thermal interface materials (TIMs) to reduce surface unevenness and contact resistance. In cold plate designs, uniform contact between the cell surface and plate is crucial, as manufacturing tolerances, pressure, and air bubbles can reduce the heat transfer coefficient and compromise safety [45]. Structural analyses identify three configurations: cooling fins between cells, tubular plates with internal channels, and partial immersion of modules in liquid. Numerical comparisons show systems with pipes or liquid “jackets” limit temperature increase with less mass penalty than extended fin systems. Complex liquid cooling systems connect to the vehicle’s HVAC system, forming multi-loop thermal architectures. A secondary battery cooling circuit links to the cabin cooling system via a heat exchanger or multi-way valve solutions that adjust flow paths based on heating and cooling needs of the package, power electronics and motor [21]. This requires balancing thermal efficiency, weight, complexity, and leakage risk, impacting spatial design and enclosure interfaces. In vehicle-level integration, direct refrigerant cooling uses cooling plates as an air conditioning evaporator, or cells are partially immersed in refrigerant. These configurations enhance temperature uniformity and reduce components, though increasing enclosure tightness and material compatibility requirements [50]. Alternative passive concepts include PCM systems, functional coatings, and composite housings with variable thermal conductivity. PCMs act as a thermal buffer, absorbing heat fluxes temporarily; however, their low conductivity, weight and volume increase and design complexity limit large-scale vehicle use [46,49]. Analysis indicates PCMs can mitigate temperature gradients but introduce thermal resistance, complicating thermal management across cycles, necessitating exploration of other passive solutions. One approach involves composite housings with enhanced conductivity, where carbon fibers or metallic elements establish “heat paths” to channel heat from hot regions to the outer surface. Studies show that these systems facilitate effective heat dissipation without active cooling, maintaining low housing weight [49]. A novel development is passive, climatically adaptive thermochromic layers on housing surfaces, adjusting optical properties with temperature. These solutions suit typical battery temperatures of 35–45 °C, operating without sensors, electronics, or active cooling, reducing HVAC load and enhancing durability [51]. Integrating these technologies shows that cooling system choice significantly influences material and enclosure architecture requirements, representing a trade-off between energy efficiency, weight and battery system complexity. Thermal management impacts cell aging, costs, and LCA outcomes, as thermal conditions determine package component durability [50,52]. Models indicate that selective cell replacement can prolong package life but increases servicing and design complexity. Literature suggests integrating batteries into vehicle composites (e.g., body panels) conserves weight and space but requires a distributed cell architecture and advanced systems to mitigate overheating and thermal runaway risks [53]. Integrated solutions also elevate service and recycling demands, affecting cost-effectiveness and thermal strategy selection.

Thermal management under different operating conditions is demanding. EV disruptors show ambient temperature affects range and cell degradation—30–40 °C increases reduce lifespan by two months. Without effective thermal management, range can drop 85% in sub-zero temperatures. Usage profiles, like acceleration and road quality, affect heat generation and cooling needs, which integrate with the package enclosure [44]. Air or cabin air cooling suits short-haul vehicles, while secondary circuits with heat pumps or AC are advanced. Direct refrigerant cooling offers better temperature uniformity and fewer components, which is crucial for varying thermal loads and limited space. System effectiveness depends on coolant temperatures, determined by heat flux, exchange surface and global heat transfer coefficient, including material properties, wall thickness and contact resistance [45]. In composite housing studies, a variable conductivity shell with carbon fibers creates “heat paths” from hot areas to the surface, enhancing conduction without coolant or extra components and maintaining low weight. BTMS reviews suggest air, liquid and PCM cooling choices should match operating profile, complexity, weight and integration with vehicle systems [54]. Muratori et al. noted that local climate and charging patterns affect electric vehicle energy balance, highlighting that BTMS design must align with charging infrastructure and grid management [55]. Life cycle analyses and tests show that real-world conditions (e.g., season, load, start-stops) and design selection impact energy use and emissions, stressing the importance of measurement data for evaluating BTMS cost-effectiveness [56]. Other studies show variable usage profiles change energy consumption, influencing decisions on complex cooling systems versus simpler ones. Simulations and control strategies should consider varying charging scenarios and carbon footprints, as these affect WTW emissions and cooling solution efficiency. Review studies suggest considering thermal management system energy use and two-zone architectures in full system simulations, as they impact vehicle efficiency [57]. Thermal analyses recommend a trade-off operating temperature range (10–30 °C) for optimizing between calendar and cyclic aging, considering cell chemistry and usage specifics. Cell production experience shows housing, tightness and interface requirements from mini-environments affect BTMS integration and costs [58]. Thus, cooling system design should factor in production and logistics, as trade-offs in service availability, weight, and temperature uniformity influence cooling architecture choice.

An additional challenge for battery enclosure systems is operation under extreme climatic conditions, particularly in cold environments. At ambient temperatures below 0 °C, and especially in the range of −10 to −20 °C, lithium-ion batteries experience a significant increase in internal resistance, resulting in reduced power output, slower charging rates, and a substantial loss of driving range. Previous studies have shown that, under severe winter conditions, vehicle range may decrease dramatically due to both electrochemical limitations and the additional energy required for battery heating. Consequently, the battery housing should not only provide mechanical protection but also contribute to maintaining a stable thermal environment around the cells. This requirement has increased interest in enclosure concepts incorporating thermal insulation layers, integrated heating elements, low-conductivity composite materials, and advanced thermal management systems capable of reducing heat losses during vehicle operation and parking [59,60,61,62]. In addition to low-temperature performance, battery housings play a critical role in preventing and mitigating thermal runaway events. Modern enclosure systems must act as effective thermal and fire barriers capable of limiting heat propagation between cells and delaying the spread of fire to adjacent vehicle components. Current research focuses on the integration of fire-resistant composite materials, intumescent coatings, ceramic barriers, phase change materials (PCMs), and multifunctional sandwich structures that combine lightweight design with enhanced fire protection [63]. Therefore, future battery housing development should simultaneously address thermal insulation for cold-climate operation and thermal runaway containment, ensuring both operational efficiency and passenger safety under a wide range of environmental conditions.

In addition to thermal runaway and low-temperature operation, battery housing materials are continuously exposed to continuous cyclic thermal loading, road-induced vibrations, and potential local electrolyte leakage resulting from cell damage. These factors may accelerate material aging, promote microcrack formation in composite matrices, degrade adhesive joints, and induce localized corrosion or chemical degradation due to electrolyte exposure, ultimately reducing long-term mechanical performance and enclosure reliability. Therefore, future battery enclosure designs should consider durability under combined thermo-mechanical and chemical exposure conditions in addition to conventional crash and thermal safety requirements.

## 4. Limitations of Conventional Metal Solutions

### 4.1. Weight Problems

The mass of traditional metal vehicle structures limits electric vehicle energy efficiency advancements. Lightweight body construction can reduce weight by 55–115 kg compared to steel; the SuperLIGHT-Car concept achieves a 101 kg reduction with optimized materials. Oversized steel structures have mass potential for battery housings. The vehicle’s mass budget, shaped by design and financial assumptions, limits offsetting heavy packages by increasing drive power or battery capacity [64]. Large battery packs’ excess weight is a major electric vehicle challenge. Research shows electric battery powertrains can be 125% heavier than combustion engines, necessitating other component weight reduction. Weight reduction cascades: less vehicle weight reduces suspension or braking system demands, further lowering accessory weight. “Mass decompounding” shows heavy metal solutions hinder secondary weight savings [11]. In enclosure design, weight directly impacts optimization processes. One method designs package housing to minimize weight, maximize vibration frequency and minimize displacements. Topological optimization and element thickness selection significantly reduce weight compared to reference variants while maintaining rigidity and strength, challenging massive, conventional metal solutions [35]. Reviews suggest enclosure weight reductions of 9–12% compared to baseline designs, enhancing performance and reducing deflection [18]. Experimental and simulation work shows parametric and topological optimization can reduce enclosure weight from kilograms to dozens without exceeding stresses, promising integration with a lightweight body [65]. Airflow gaps and thermal analysis keep cell temperatures within limits, aligning thermal and mechanical needs. Maintaining heavy cases affects more than component weight. For electric vehicles, a 10% weight reduction can boost range by 13.7%, showing energy efficiency’s sensitivity to weight [11]. Predominantly steel-based battery housings need larger packages to maintain range or accept reduced autonomy, increasing demand for battery materials and emissions. Well-designed lightweight hybrid or electric vehicle bodies can match internal combustion vehicles through strategic mass management and metal structure reduction [64]. Weight impacts not only components but also the body and crash system architecture. Research on side impact highlights that a heavy floor package requires substantial reinforcement of thresholds and the floor structure, as the faucet must remain undeformed, necessitating massive, thin-walled steel or aluminum profiles, often reinforced with extruded profiles, exacerbating weight in the floor zone [38]. A lighter, rigid battery cassette could distribute load-bearing and energy-intensive functions among body components, reducing oversized metal components. Literature suggests flame-retardant polymer compounds in module frames, latches and top covers can create thinner, lighter components while maintaining thermal and impact resistance. PPE with glass amplifier, ABS, or PC/PBT composites are practical solutions [66]. However, the polymer approach may introduce cost and safety challenges in large-scale production. Findings indicate that traditional metal solutions’ weight affects the entire dependency chain: from other components’ sizing to leveraging secondary weight savings, range and emissions throughout the vehicle’s life cycle [11,18,35,64].

### 4.2. Corrosion Resistance Issues

Corrosion considerations critically influence the suitability of traditional metals for battery housings. In steel structures, corrosion is a primary design constraint. Steel housings require protective coatings, as seen in the Chevrolet Bolt EV chassis with an anti-corrosion system. A plastic coating is recommended for the underside to reduce dirt and moisture, minimizing corrosion. Steel concepts need painting or metallic coating; electroplating is an example [35]. This shows corrosion resistance in steel systems is complex but aluminum and multi-material systems also face challenges. Research suggests that aluminum can replace steel if galvanic corrosion is minimized. Connections must prevent galvanic cells between aluminum and other metals [45]. Aluminum alloys have good corrosion resistance due to their passive layer, but barrier coatings are crucial, especially regarding road salts [1]. Another example is magnesium alloys, but they often also face corrosion issues, highlighting corrosion resistance’s importance in material selection. Corrosion issues affect electrical connections and contacts. Battery component analysis shows atmospheric and galvanic corrosion at metal interfaces and fretting corrosion in vibrating joints as key degradation mechanisms. These increase electrical resistance, heat at contacts, degradation and safety hazards. Thermal expansion and dynamic loads with corrosive effects in joint areas are crucial [67]. In aircraft, casing damage and contact corrosion can disrupt venting and thermal conductivity, promoting faster thermal events at low pressure [67]. Literature on battery safety in aviation emphasizes venting categorization and notes that standard tests may not reflect the behavior of large cell packs, affecting corrosion protection design. Corona discharge at high voltage and its interaction with damaged connections require attention during certification and studies [68]. Corrosion protection complements strategies for enhancing mechanical strength, ensuring tightness, and inhibiting failure propagation. Fiber-reinforced polymer composites offer significant corrosion resistance advantages over metal housings. Analyses of lightweight materials for electromobility highlight CFRP and GFRP’s high resistance to environmental corrosion, thereby reducing the need for intensive protective coatings [2]. Research on composite battery housings shows fiber composites have excellent corrosion resistance, crucial due to corrosive emissions and exposure to aggressive environments [69,70]. This property maintains package protection without compromising housing material over long service lives. Findings suggest traditional steel and aluminum require complex corrosion protection measures, while fiber composites reduce degradation risk. However, corrosion challenges persist in metallic bonding zones and high-voltage components [35,69,70].

### 4.3. Impact on Energy Efficiency

The proportion of heavy steel and aluminum in vehicle structures affects energy demand. Replacing steel with aluminum can reduce weight by 11–25%, improving energy efficiency. However, aluminum production is more energy- and carbon-intensive than steel due to bauxite and electrolysis processes and emissions. Life cycle analyses highlight the need to balance lightweight benefits with production energy inputs, with aluminum metallurgy’s energy source being crucial [71]. Structural battery composites show that replacing steel and aluminum with multifunctional structures can cut energy use in an electric vehicle’s life cycle. A study showed that replacing steel and aluminum panels with composites reduced weight from 41 kg to 14 kg while maintaining rigidity, decreasing the NMC111 package by 7 kg, thus lowering energy use through avoided electricity and battery production. The energy reduction value (ERV) was 0.069 Wh/(kg·km), showing the traction energy consumption’s sensitivity to weight. Heavy metal housings limit secondary battery weight reduction, as lower body weight allows a lower capacity package while maintaining range. Results suggest energy benefits from lighter structures are linked with avoided lithium-ion battery production, a key part of an electric vehicle’s energy footprint [34]. Steel from electric arc furnaces (EAFs) has 54% lower emissions per unit weight than steel from primary materials due to reduced energy use and more non-fossil energy. A significant portion of automotive steel is recycled in an open loop for non-automotive uses, limiting closed-loop benefits in this sector. Increasing recycled steel in vehicles can reduce steel production energy costs but does not address heavy enclosures affecting operational energy [71]. In a circular economy, addressing energy efficiency is crucial. First, reduce embedded energy by using more recycled steel and aluminum, optimizing metallurgical processes and selecting aluminum production regions based on energy mix. Second, reduce inactive component weight, like package housings, to lower traction energy demand and battery production scale, as shown for structural composites. Scenario analyses show transitioning from conventional steel–aluminum to structural battery composites can significantly reduce life cycle energy consumption and climate impact of electric vehicles, despite higher composite manufacturing energy costs. This reduction is due to decreased vehicle weight, reduced operational energy consumption and eliminating battery pack production. Consequently, heavy metal enclosures are inefficient over the life cycle, even when partially recycled [34,71]. Table 5 shows a comparison of the properties of EV battery housing materials.

## 5. Composites as Lightweight and Multifunctional Alternatives

### 5.1. Polymer Composites

As mentioned briefly previously in e-mobility applications, polymer composites include CFRP, GFRP, natural fiber, and hybrid composites. For battery chambers, high strength-to-weight ratios, corrosion resistance, and thermal and electromagnetic functions are crucial. CFRP offers high rigidity and strength for weight reduction, while GFRP is economical with good tensile and impact strength, used in battery housings. Natural fiber composites are lightweight and renewable but have lower strength and moisture issues. Hybrid composites balance cost, weight and mechanical properties [9]. Lightweight polymer composites are used in frames, housings, and panels. In battery chambers, CFRP, GFRP, and hybrids provide thermal insulation, fire resistance, and shielding, reducing metal weight. Key factors include thermal stability, EMI shielding and fire resistance. Natural and hybrid composite suit interiors prioritize weight, aesthetics, and sustainability [3]. Polymer composite selection is tied to processing techniques. In e-mobility, RTM molding, pressing, pultrusion, and additive manufacturing are used. RTM allows precise fiber placement for structural parts. SMC and BMC pressing enables rapid cycles for large panels. Pultrusion produces fixed cross-section elements cost-effectively. For prototypes, 3D printing offers design freedom, whichis useful in battery module carriers. Literature highlights biofibers and biodegradable or bio-based polymer matrices in electric vehicles. Research of biocomposites highlights benefits like higher impact absorption energy, easier processing, and lower health costs compared to glass fibers. Manufacturing requires less energy, reducing the environmental footprint. Efforts focus on enhancing moisture resistance, property repeatability and biofiber integration in pressing and RTM processes to expand applications from interior elements to semi-structural parts [3,9,39,72,73]. Recycling and circular economy integration remain significant for polymer composites, aligning with previous conclusions. Lightweight composites, despite recycling challenges, can reduce life cycle energy consumption due to weight reduction and decreased electricity and battery component production, especially with bio-based materials. Model studies show integral battery housing and changes in load-bearing rigidity affect force distribution and energy absorption during collisions, suggesting composite housings as active absorption elements [74]. This requires considering trade-offs between weight, body connection, and deformation resistance. Table 6 presents a comparison between processing, used materials and a brief description of manufacturing technologies.

Carbon fiber-reinforced polymer (CFRP) composites, known for their high strength-to-weight ratio, rigidity and resistance to corrosion and fatigue, are increasingly used in e-mobility to reduce weight while maintaining structural integrity. These composites are employed in automotive components like chassis, roof panels, suspension arms, and battery housings, where weight reduction enhances vehicle range and performance. In electric vehicles, CFRP replaces traditional materials like steel and aluminum, offering significant weight savings. For instance, BMW uses carbon fiber for lightweight vehicle bodies, while others use it in suspension and powertrain components, achieving 30–60% weight reduction compared to metals. Glass fiber-reinforced polymer (GFRP) battery housings provide necessary strength and rigidity with reduced weight [75]. CFRP’s structural properties depend on the die type used. Thermosetting matrices, like epoxies, form stable cross-links that do not remelt, while thermoplastics can be reshaped, offering a balance between stiffness and flexibility. These composites are cost-effective to manufacture due to simple processes, and their properties can be tailored with fillers and additives [5]. CFRPs are fabricated using techniques like resin transfer molding (RTM), thermosetting and thermoplastic pressing, and additive manufacturing, especially in the automotive sector. RTM allows precise fiber arrangement in molds, creating complex, high-quality geometries for thin-walled battery housings and load-bearing components. Pressing techniques enable short cycle times for large panels, while additive manufacturing produces lighter, optimized shapes, like battery module carriers and components integrating structural functions with cable routing [9]. Material trend analyses show the rising use of thermoplastic composites with carbon or glass fibers in the automotive industry. These are used in semi-structural components and interiors, offering shorter processing times, recyclability and high mechanical strength. Laminates of carbon fibers and a suitable matrix create structural panels with steel-like rigidity, reducing weight, as seen in composite roof panels and suspension elements [75]. Sustainability research explores carbon fibers in hybrid and bio-composites. Studies on electric vehicles indicate that recycled carbon fibers and composites with bio-based matrices reduce manufacturing energy use while maximizing weight reduction. However, recycling technologies for thermosetting composites pose challenges for the circular economy, despite their operational energy savings [34,75]. The economic viability of expensive lightweight materials depends on battery costs; process cost analyses suggest that as traction package prices drop, the economic benefit of lightweight materials may decline [76]. Thus, vehicle weight reduction strategies involve balancing higher material and processing costs with the potential to reduce battery capacity and operating expenses.

### 5.2. Advantages of Composites in Battery Housings

Utilizing composites for battery housings significantly reduces weight while maintaining or enhancing mechanical properties. Carbon and glass fiber composites achieve up to a 56% weight reduction compared to steel, offering high specific strength and energy absorption. In the e-mobility sector, CFRPs exhibit a high strength-to-weight ratio, while GFRPs, though heavier, still reduce component weight and preserve load capacity [2,75]. Composite sandwich structures, with high-strength cladding and a lightweight core, provide high flexural rigidity and energy absorption at low density, making them mass and cost-effective for low-to-medium flexural stiffness applications. This allows for thin-walled, rigid plates replacing solid steel or aluminum sheets. Advanced composite systems include CFRP hybrids with metal components and fiber-to-metal laminates, integrating CFRP rigidity with aluminum or steel stiffeners. “Smart Steel” laminates with a polymer core denser than aluminum achieve up to 35% weight savings while maintaining thickness and bending stiffness, offering alternatives to monolithic steel sheets for robust load-bearing [11]. Sandwich structures with foam or honeycomb cores and CFRP cladding enhance energy absorption while maintaining low density, which is advantageous for crash zones [32]. Composite manufacturing technologies streamline chassis design. RTM and VARTM processes produce thin-walled, complex components with high fiber content, resulting in high rigidity with minimal material use. Additionally, 3D printing with thermoplastic composites, including continuous fibers, strategically places material only where necessary, reducing waste and component weight compared to milled or stamped solutions. Reducing chassis weight improves energy efficiency significantly [1,9,34]. Advanced composites, despite higher production energy costs, have lower energy consumption over the life cycle due to weight savings compared to metals. Weight reduction with composites is due to the high specific strength of CFRP and GFRP, efficient sandwich solutions, advanced molding processes and optimized geometries for stiffness and collision resistance. The battery pack includes components like busbars, cooling, and management systems, affecting energy density and potentially limiting lightweight enclosure benefits. Literature indicates that cell format variations, aging mechanisms, and recycling issues may affect scalability and cost-effectiveness, necessitating aging models and life-cycle analyses in design [37,77].

The second most important advantage is corrosion resistance. Reinforcing fibers and polymer matrix prevent classic electrochemical corrosion seen in steel and aluminum. In automotive and e-mobility sectors, fiber-reinforced polymers (FRPs) offer high strength and corrosion resistance, beneficial in humid and saline environments [9]. Composite enclosures avoid perforation corrosion, maintaining protection even with paint damage, unlike metals [69]. Nanocomposite coatings significantly enhance corrosion resistance. Studies show polymer-reduced graphene oxide (rGO) coatings on substrates improve interfacial strength, stability in water, and surface resistance with minimal weight increase over metallic laminates. Water immersion tests show that lower rGO content maintains coating continuity without particle loss after 48 h, indicating moisture resistance [2]. In EV technology, carbon and glass fiber-reinforced polymers provide high corrosion resistance and a good strength-to-weight ratio, improving energy efficiency and durability. Fiber composites, unlike steel, do not form local corrosion foci, maintaining mechanical properties despite moisture or road salts. CFRPs also resist fatigue under cyclic loading and corrosive conditions, which is vital for housings in vibrating and fluctuating temperatures in EVs [69]. Developing recyclable-compatible coatings advances corrosion resistance and supports the circular economy. Research suggests replacing traditional matrices with bio-based or thermoplastic resins and analyzing polymer-rGO systems in steel and aluminum housings for energy savings, emission reductions, and material recovery. This suggests graphene composites and coatings enhance enclosure durability while reducing reliance on processed metallic coatings and associated environmental impacts [2].

### 5.3. Potential Drawbacks and Limitations

In composite battery housings, material and manufacturing costs challenge widespread adoption. Carbon fibers’ high cost limits their use in mass-produced vehicles despite their benefits. CFRP components’ production involves expensive materials, complex preform preparation, and precise molding, raising costs. While glass and natural composites are cheaper, achieving quality and functional integration often requires advanced technologies, increasing costs. Lightweight composites for e-mobility must compete with metal solutions from established supply chains. High-pressure RTMs, high-speed curing, and automated fiber laying reduce cycle times but need costly tooling and infrastructure, limiting high-volume vehicle segment scaling [1,9]. Lack of standardized testing hinders composite cost-performance comparison, delaying affordable composite adoption. Optimizing processes like additive techniques, 3D printing, and nanotechnology could cut costs, but unreliable long-term data may increase uncertainty and deter investment [78]. Manufacturing processes greatly influence total costs. E-mobility technological reviews identify RTM, pressing, pultrusion, and 3D printing as key methods for structural components. RTM and compression molding offer high quality and short cycle times, but require costly molds and precise resin systems. The autoclave provides superior performance but is too expensive for most automotive applications, mainly used in aerospace, limiting its use for serial battery housings [39]. Research on lightweight composites shows additive technologies and automated fiber laying are still scaling, with cost risks for vehicle manufacturers due to the gap between demonstrators and high-volume production [9]. Composite enclosure costs should be evaluated from a life cycle perspective. Although manufacturing advanced composites is more energy-intensive than steel, reducing vehicle weight decreases operational energy use and battery pack size [34]. Economic challenges also exist at the end of life. Recycling, especially thermosetting composites, is limited, creating uncertainty about future waste management costs and residual value. Studies stress developing efficient, cost-effective recycling for wider automotive industry use, as the current lack of mature recycling infrastructure poses economic risks [79].

The use of composites and polymer plastics in battery housings challenges efficient, cost-effective recycling. Thermoplastics in modules, housing, cell holders and energy-absorbing elements are lightweight and robust, sometimes with flame retardants, complicating recycling into original materials. This hinders material cycle closure, package repair, and cell reuse. Separating components in composites or thermoplastics is complex. Strong adhesives in package designs make mechanical disassembly inefficient, blocking valuable material recovery. Chemical baths swell adhesives beyond bonding forces to separate cells from glued structures, adding process steps and new chemicals, raising environmental and cost concerns [80]. Recycling complex products requires multi-step processes combining mechanical, thermal, and chemical operations. For complex waste streams, a single technology rarely achieves high recovery efficiency. Thus, grinding, mechanical separation, pyrometallurgy and hydrometallurgy are used for metallic fractions. However, effectiveness is limited by the weakest link, often the collection or pre-processing stage, where valuable materials are lost. The loss of metals to unsuitable fractions or landfills and contaminants during processing worsens recycling’s environmental and economic balance. Composite materials in enclosures increase waste stream heterogeneity, complicating effective recovery design [81,82]. For traction batteries, recycling is limited by non-standardized package designs, complicating dismantling. Shredding entire packages benefits small electronics but yields lower purity and value when scaled to large traction packages. Mixing composites, plastics, and metals in shredding creates high impurity fractions, requiring intensive hydrometallurgical treatment and generating by-waste. High-quality cathode or anode fluxes need additional delamination and binder neutralization [82]. New recycling proposals for polymer composites and barriers lack verified environmental profiles, creating uncertainty about life-cycle benefits and circular economy integration. Systemic barriers in composite enclosure recycling stem from design limitations and lack of standardized battery protocols. Analyses suggest current traction battery designs hinder dismantling. Non-uniform EoL process requirements elevate recycling costs and complicate waste management. Lack of standardized dismantling, testing, and classifying EoL batteries extends pre-recycling, increases material stream uncertainty, and reduces recovery profitability. Thus, composite enclosures with potential advantages operate in a system unfit for efficient recovery [83].

## 6. Challenges of the Circular Economy

### 6.1. Identification of Reusable Raw Materials

Identifying reusable raw materials requires examining the entire battery and housing value chain, including material data, product architecture, and recycling and reuse technologies. A key premise is ensuring information flow on battery material composition throughout its life cycle, primarily via a “battery passport.” This passport tracks material flows and battery conditions, aiding end-of-life decisions [81]. Similarly, “circularity passports” integrate materials tracking with digital infrastructure, providing data on usage history for reuse, remanufacture, or recycling decisions [84]. For traction modules, digital passports with QR codes and health registers store data on origin, state of health, and refurbishing history, relevant for selecting units for a second life [85]. Effective raw material identification also requires access to cell chemistry and package configuration data during sorting. Digital EV battery passports with component dimensions, 3D CAD models, and degradation levels aim to automate testing, disassembly, and sorting, assigning units to reuse or recycling streams. Sensor and AI/ML-based sorting systems recognize geometries and fasteners, enhancing high-value component location during automated disassembly. The lack of universal standards for package design and labeling complicates identification and sorting; however, module standardization and digital labeling (e.g., QR codes) could improve precision in extracting critical raw materials. From a circular economy perspective, functional metals, cathodes, and anodes (Li, Ni, Co, Mn, Cu, graphite) are prioritized, yet identifying reusable components should also include structural components like aluminum and steel housings [81]. Designing product architecture to repurpose modules after the first life cycle is recommended, necessitating modules as functional units for dismantling, testing, diagnosing, and reconfiguring first and second-cycle modalities [45]. Research on upcycling EV modules for heat storage emphasizes preserving metal module housings as “cassettes” for phase change material, identifying them as components with high reusability value requiring information on geometry, mechanical integrity, and potential PCM integration [22]. Analyses of batteries’ second life in stationary applications suggest their use in energy storage for buildings with PV installations can yield energy and economic savings and extend storage system life. This potential highlights the need for efficient parameter tracking and unified testing to ensure interoperability and safety [86]. Identifying reusable materials and components is influenced by regulations and return chain organization. For example, Japanese regulations require manufacturers to collect and recycle lithium-ion batteries, with annual reporting on collection and recovery rates. This demands systems for tracking return streams. In California, “core exchange” and “producer takeback” strategies divide battery management between the dismantler and manufacturer, requiring assessment of battery condition for reuse or recycling [80]. Establishing logistics infrastructure and dismantling centers to extract high-value components ensures quality recycling. Policies support digital tools for optimizing end-of-life battery scenarios, logistics, dismantling networks and battery passports to enhance material flow transparency. Such systems are crucial for identifying reusable materials, including cell active materials and enclosure components for second-life applications [55,80,81,85,87].

### 6.2. Composite Recycling Technologies

In the field of recycling, four main groups of processes can be distinguished which include: mechanical, chemical recycling, energy recovery and landfill. Although several recycling routes have been proposed for composite materials used in EV battery housings, their practical implementation differs significantly in terms of technological maturity, recovery efficiency, economic attractiveness, and industrial scalability. Therefore, a comparative overview of the most relevant recycling pathways is presented in Table 7.

As shown in Table 7, mechanical recycling currently represents the most mature and economically attractive solution for large-scale industrial implementation. In contrast, pyrolysis and solvolysis allow the recovery of higher-value carbon fibers and provide greater circularity potential, although their wider adoption remains limited by processing costs and infrastructure requirements. Consequently, future EV battery housing systems should increasingly consider recyclable thermoplastic composite materials and design-for-recycling principles to improve both environmental and economic performance throughout the product life cycle.

Although recycling technologies enable the recovery of valuable reinforcement materials, recycled fibers cannot generally be considered equivalent substitutes for virgin fibers in high-performance structural applications. Mechanical recycling results in substantial fiber shortening and matrix contamination, whereas thermal recycling processes may alter fiber surface chemistry and fiber–matrix interfacial properties. Consequently, reductions in tensile strength, stiffness and impact resistance are commonly reported, limiting the reuse of recycled fibers in primary load-bearing battery enclosure structures. In many cases, recovered fibers are therefore redirected towards secondary or semi-structural applications, illustrating the continuing challenge of achieving true closed-loop recycling of advanced composite battery housings.

#### 6.2.1. Mechanical Processes

The mechanical processing of used lithium-ion batteries is crucial for recycling, enabling the recovery of active materials and structural components. Processes like shredding, crushing and grinding open cells to produce materials where components are liberated for separation into distinct fractions [82,85]. This involves two stages. Initially, low-speed shredders safely open cells and pre-grind the package, with safety measures to minimize thermal runaway risks, such as using a gaseous atmosphere, water spraying, or ventilation. Pre-discharging cells is recommended to limit energy release during shredding short circuits [82]. The second stage uses impact mills to further comminute electrodes, detaching active material from metal film for fraction separation. After comminution, physical separation operations like sieving, air classification, magnetic separation and eddy current separation are employed. Differences in density, particle size, and magnetic properties separate ferrous metals, non-ferrous metals (e.g., aluminum, copper), plastics, and other components from the electrode powder mixture, known as the black mass. These operations yield a powder rich in Li, Co, Ni, and Mn for hydrometallurgical metal recovery, while metal and polymer fractions are directed to appropriate recycling or energy recovery streams [85]. Industrial mechanical processing lines involve multi-stage crushing, screening, and magnetic separation in an inert or controlled atmosphere. For example, plants with two-stage crushing and air and magnetic separation can separate steel, aluminum, black mass, non-ferrous metals, and plastics. Some technologies integrate this with electrolyte recovery through evaporation and condensation, reducing solvent content in solid streams [88]. Mechanical pre-treatment can enhance the economic efficiency of downstream processes, as improved separation of early fractions reduces contamination of materials for thermal and chemical treatment. Mechanical processes for cell opening have limitations. In large-traction packages, diverse material mixing during shredding lowers purity and value, promoting down-cycling. Mechanical crushing in an inert atmosphere produces a black mass with binders, coal and fine aluminum and copper particles, requiring intensive processing and reducing quality. Lack of package design standardization and structural adhesives complicates manual disassembly and electrode separation, increasing energy consumption and material loss risks [81,82,88].

#### 6.2.2. Chemical Processes

The chemical processing of spent lithium-ion batteries mainly uses hydrometallurgical techniques to selectively dissolve metals after mechanical processing. Following comminution and physical separation, a black mass enriched with cathode and anode metals is subjected to acid or alkaline leaching. Appropriate reagents, temperature and concentration ensure high dissolution efficiency [82,88]. Further purification involves impurity removal, solvent extraction and precipitation. Initially, undesirable components like iron are precipitated through oxidation and pH adjustment to Goethite FeOOH. After reducing, iron, copper, cobalt and nickel are selectively separated using solvent extraction systems chosen for metal ion affinity. Depending on parameters, sulfate salts of Cu, Co, and Ni, or suitable carbonates and chlorides, are obtained. This process achieves high metal yields with purity levels suitable for battery reuse. European facilities, such as those in Sweden and Finland, integrate these processes with black mass recycling, achieving up to 95% metal recovery and producing industrial-grade cathode precursors. Hydrometallurgy also allows multi-year recovery of certain solvents and by-products, reducing secondary waste [88].

Interest in recovering electrolytes, traditionally incinerated or decomposed in pyro- and hydrometallurgical processes, is growing due to their low economic value and toxicity. Recovery is crucial for safety and reducing greenhouse gas emissions. Three methods are outlined: solvent extraction, vacuum evaporation, and supercritical CO_2_ extraction [89]. Supercritical CO_2_ extraction is detailed for recovering carbonate solvents and LiPF salts. Response surface method tests show operating pressure is key; at 23 MPa, 40 °C and 45 min, 85.07% extraction efficiency was achieved for organic carbonate-based electrolytes. Studies confirmed pressure- and temperature-dependent polarity of supercritical CO_2_ influences efficiency, with linear carbonates (DMC, EMC) more effectively recovered than cyclic carbonates (EC, VC). A multi-step closed-loop recycling process was developed, combining supercritical CO_2_ extraction, purification using ion exchange resins and molecular sieves, and replenishment of missing components. After removing HF and water to industry standards and reconstructing the composition, the recovered electrolyte achieved ionic conductivity of 0.19 mS/cm at 20 °C and operated in a Li/LiCoO half-cell with an initial capacity of 115 mAh/g and retention of 66% after 100 cycles at 0.2C. These findings show that chemical electrolyte recycling can yield a product comparable to virgin material [89]. The amount and composition of the electrolyte affect degradation mechanisms and recovery possibilities. Studies on Li–S cells indicate that excess electrolyte and specific formulations can obscure degradation mechanisms and alter relationships between components that are easier to extract, complicating reconstruction of the original electrolyte composition [90]. Extraction and purification parameters should consider not only the chemistry of individual compounds but also the original amount and distribution of electrolyte within the cell structure.

Attention is directed towards the challenges of end-of-life (EoL) materials, particularly LiPF degradation in moisture, forming toxic HF and organo(fluoro)phosphates. This requires stringent process control and improved extraction and purification technologies [82]. In circular economy development, advanced hydrometallurgical processes with specialized electrolyte recovery techniques are promising for recycling multi-metal traction batteries [82,88,89]. Automating dismantling processes, including battery packs, is crucial for recycling and remanufacturing. Life-cycle analyses show that in high-throughput scenarios, material production impacts the environment, with cobalt and nickel extraction significantly contributing to GHG and SOx emissions. Integrating chemical and hydrometallurgical recovery with dismantling automation can improve energy efficiency and reduce emissions, especially in cobalt and nickel-dependent supply chains [91,92]. In non-aqueous electrochemical systems, LiPF salts and carbonate mixtures affect solution polarity and conductivity, complicating EoL battery electrolyte recovery, a factor in designing regeneration procedures [48]. Adapting solutions from non-aqueous redox flow battery research may require purification steps or specific modifications during electrolyte reconstruction. As structural batteries require specific material forms and functions, compounds from recycling may need transformation for compatibility with electrochemical-mechanical composite manufacturing processes [73]. Regeneration should include chemical recovery and transformations to align material properties with techniques like fiber coating or integration with polymer-ceramic matrices.

### 6.3. Design for Disassembly

Designing battery enclosures for disassembly aligns with the design for disassembly (DfD) paradigm, facilitating repair, reuse, remanufacturing and recycling of components. DfD guidelines advocate modular architecture, fewer components, recyclable materials, avoiding hazardous substances, and using accessible, detachable connectors instead of adhesives. Emphasis is on lightweight components that do not require special tools and are not coated, which is crucial for battery enclosures due to mechanical and electrical integration and safety requirements. Disassembly complements should be designed for recycling (DfR) rather than reversing it. Cost-effective assembly solutions can hinder disassembly, so “separability” of joints is analyzed, minimizing inseparable adhesives and selecting bonding techniques to improve recyclability. DfD research shows disassembly trials reveal connector selection’s impact on material recovery, informing new designs. In battery systems, modular architecture for various end-of-life scenarios is emphasized, grouping components by life cycles to increase reuse, e.g., separating ‘core’ from replaceable ‘peripheral’. For battery packs, this means designing modules as units that can be tested, replaced, and reconfigured without damaging the chassis. Implementations show a hierarchical BMS architecture with cell- and module-level monitoring allows for cell history recording, facilitating second life assessment and safe service. This increases electronic complexity, affecting disassembly and cost, but avoiding detachable connections is often impossible, especially for safety-required permanent connections. Design methods are needed to “reuse” non-separable joints, e.g., predictably cracking during shredding. From a circular economy perspective, integrating DfD with dematerialization, modularity, and sustainability is emphasized. Designing with minimal materials and optimal combinations can enhance recycling efficiency and reduce hard-to-recycle fractions. Durable enclosures that resist fatigue, degradation, and corrosion can slow raw material needs when paired with service and repair programs [84]. Designers must balance functional requirements, costs, and dismantling needs, as material and construction choices affect future raw material recovery [45,93]. Design for dismantling includes technologies like automated disassembly with vision and machine learning, human-assisted robotic workstations, and reversible connections that do not hinder miniaturization. Composite materials require connectors for selective disconnection under specific conditions. Effective Design for Dismantling (DfD) in traction batteries requires integrating these technologies with product architecture and dismantling priorities, such as isolating hazardous materials and extracting high-value components needing dedicated process chains [45,93].

## 7. Material Recycling Paths

### 7.1. Metal Recycling

#### 7.1.1. Aluminum

Aluminum is crucial in metal recycling discussions due to its energy-saving potential in secondary production. “Sustainable aluminum” is a fully recycled alloy needing about 95% less energy than primary production while maintaining mechanical properties. It reduces CO_2_ emissions and raw material demand, aligning with circular economy principles. Secondary aluminum production involves smelting scrap from various industries, sorted by alloy type using techniques like magnetic separation, air separation, and manual sorting. Surface contaminants are removed with centrifuges or coating removal equipment, enhancing remelting energy efficiency and reducing emissions. Secondary aluminum requires less than 10% of the energy of primary production, achieving a low environmental footprint [94]. Recycling post-consumer aluminum scrap significantly reduces emissions, involving fraction separation, melting, refining and purification. Effective liquid metal quality management is crucial for casting durability and mechanical properties; reducing oxide impurities and non-metallic inclusions is essential [94,95]. A challenge in recycling aluminum is liquid metal contamination with hydrogen and oxygen, causing porosity and ductility issues. Advanced purification technologies like rotational inert gas introduction and salt fluxes help reduce impurities. Ultrasonic treatment shows promise for removing gas bubbles but is limited to smaller volumes. Intensive liquid metal shear is more efficient for reducing porosity and improving plasticity than traditional degassing. The structure and composition of scrap significantly influence its reuse in alloys with high mechanical, fatigue, and corrosion requirements. Typical secondary mixtures (twitch) have varying Si, Fe, Cu, Zn, and Mg content, challenging stringent material specifications for critical load components. Electrification projections indicate increased scrap from rolled products and dismantled electric vehicles, potentially worsening surpluses of secondary alloys with high trace elements. Research suggestions include finding new applications for sink alloys in electromobility, improving impurity tolerance through microstructure control and developing technologies for sorting and purifying liquid metal to manage diverse scrap mixtures [95]. Life-cycle analysis shows using aluminum from electric arc furnaces (EAFs) and post-consumer scrap significantly reduces greenhouse gas emissions compared to primary production. However, the lack of alloy-specific recycling and mixing of multiple scrap grades leads to downcycling to low-value casting alloys. From a circular economy view, it is advisable to increase scrap share in production and develop precision sorting technologies to maintain alloy quality for modern electric vehicle bodies and housings [71,95]. Recycling EV component analyses reveal inaccurate material separation before melting limits metal recovery and degrades secondary alloys, worsening downcycling [96]. Improving demolition and separation processes, especially removing non-magnetic contaminants and non-ferrous metals, is crucial for preserving aluminum’s value and reducing emissions. Developing non-thermal, electric systems for fuel production from atmospheric CO and declining renewable energy costs may reduce emissions in the aluminum recycling chain. Such installations operate at room temperature and adapt to intermittent power supply, using inexpensive surplus energy to power EAF furnaces and liquid metal purification equipment [8]. However, practical emission reductions likely depend on integrating renewable infrastructure with recycling plants and investing in network and logistics to use flexible, low-carbon energy sources without compromising efficiency.

#### 7.1.2. Steel

Steel remains vital in automotive structures due to its ferromagnetic properties, facilitating efficient recycling. All steel grades can be smelted together, unlike aluminum, which requires grade separation before smelting [11]. Recycling steel in electric arc furnaces (EAFs) cuts greenhouse gas emissions by 54% per unit weight compared to virgin steel production, thanks to reduced power consumption and increased non-fossil energy use [71]. EAF production requires 9–12.5 GJ/t of crude steel, while blast furnaces consume 28–31 GJ/t, offering substantial energy savings. EAF steel use is crucial for sustainability in the automotive sector [11,71]. Despite high recycling rates, much automotive steel is recycled in an open cycle, often ending in concrete reinforcement, losing its original alloy function. Shredding and secondary smelting mix grades, with impurities like copper or tin degrading secondary steel quality. Japanese market studies suggest that improved dismantling and sorting of steel products can cut alloying element needs in EAFs by 10% and reduce alloy additive emissions by up to 28%, but detailed sorting by alloy composition is essential [12]. End-of-life vehicle processes involve dismantling and shredding, with ferrous and non-ferrous scrap separated through magnetic and mechanical means. The U.S. scrap industry supplies 12–18 million tons of short iron and non-ferrous scrap annually, generating energy savings [71]. However, many non-metallic materials, like plastics, are relegated to ASR, typically incinerated or landfilled, highlighting recovery strategy limitations [87]. Recycling technology limitations in battery literature show pyrometallurgical processes often fail to recover materials like graphite, aluminum and lithium, requiring combined pyrometallurgical and hydrometallurgical steps, complicating modeling and environmental impact allocation [97]. Findings suggest the steel industry should explore hybrid processing and precise sorting procedures instead of relying solely on melting mixed scrap. Applying circular economy principles to automotive steel involves two strategies: increasing recycled steel from electric arc furnace (EAF) processes in new vehicles to reduce energy and emissions and enhancing scrap quality through advanced disassembly and sorting to minimize downcycling and maintain high-strength alloys. Analyses highlight that, although steel is the most recycled material by weight, realizing its circular potential requires transitioning from melting mixed scrap to selective recycling pathways and vehicle designs that enable easier dismantling and alloy segregation [12,71].

### 7.2. Recycling Composites

Fiber recovery from carbon fiber reinforced composites is crucial for reducing their environmental impact. Life cycle analyses show fiber recovery from CFRP composites can significantly reduce environmental impacts, with mechanical, thermal and chemical methods explored. In structural and energy storage composites, carbon fibers act as carriers and electrodes, increasing material value. Solvolysis, a chemical process, is a promising recovery method, decomposing the polymer matrix in the liquid phase to separate carbon fibers while preserving their length and properties. Studies on traditional CFRP composites show chemical fiber recovery retains fiber strength and length, which are essential for reuse in structural applications. A similar approach could apply to electrode carbon fibers in structural battery composites, though electrolyte presence and lithium content effects need further study. Thermal techniques, particularly pyrolysis, are another method. For conventional CFRP composites, pyrolytic fiber recovery is more technologically advanced than other methods [98]. Pyrolysis occurs anaerobically, decomposing the organic matrix without oxidizing carbon fibers; resulting gases and oils can be used for energy [88]. However, structural analyses of battery composites show a lack of data on electrolyte and cyclic lithium effects on fiber stability during pyrolysis, indicating a research gap [34]. Recycling CFRP composites in e-mobility highlights fiber recovery from post-consumer waste, including end-of-life aircraft. By 2030, 6000 to 8000 retired aircraft in the U.S. and Europe are expected to generate about 3000 tons of recycled carbon fiber annually, already used in the automotive sector as cost-effective, high-strength reinforcement. This secondary fiber stream reduces costs and emissions of virgin carbon fiber production and supports lightweight components in electric vehicles [75]. Future advancements in structural battery composites are exploring fiber recovery integration with process innovations. Bio-based fibers like lignin and microwave heating in fiber production could cut energy use by 90% compared to traditional pyrolysis. Developing recovery technologies and new virgin fiber production pathways is vital for composite environmental balance, as recycled and bio-based fibers can reduce fossil raw material demand. Life-cycle analyses authors stress fiber recycling from composites as a key “technological development direction” to evaluate alongside bio-based precursors or new manufacturing methods for assessing long-term environmental benefits of composites in electric vehicles. With increased secondary carbon fiber supply from aerospace and industrial CFRP pyrolysis recycling lines, this supports a more circular material system for lightweight battery enclosures [34,75,88].

The recovery of the polymer matrix from composites used in battery enclosures is less advanced than fiber recovery but is crucial for sustainability and circular economy design. In CFRP and GFRP composites within e-mobility, research mainly focuses on fiber recovery, while the polymer matrix is often burned or decomposed thermally, hindering material cycle closure [34]. Composites include polymer, ceramic and metal matrices. In traditional FRPs, polymer matrices can be thermosetting (epoxy, polyester, vinyl ester) or thermoplastic, with the latter allowing recycling through melting and re-molding, avoiding energy-intensive chemical decomposition. PMCs are cost-effective due to simple manufacturing and modifiable properties with fillers and additives, important for future recovery and reuse [5]. A promising approach to preserving the polymer matrix is polymer–rGO coatings on composite housings and laminates. Research focuses on coating and substrate composition (PA, PE, carbon fabrics) and their mechanical, barrier, and interfacial properties. Stable multilayer systems are designed with high tensile strength, low permeability, and excellent adhesion to withstand moisture, thermal cycling, and electrolyte vapors. Endurance, immersion, and tear tests evaluate the coating–substrate interface’s durability, vital for long-term barrier function, complicating mechanical recycling due to layer separation. An extension is developing PA/PE laminates with rGO coating as metal-free alternatives to aluminum laminates. This approach aims to retain the polymer matrix and barrier layer long-term, with life-cycle analysis comparing energy savings, emission reductions, and recyclability to metallic solutions. LCA analyses are anticipated for polymer–rGO systems on steel or aluminum substrates to assess if energy and emission savings outweigh the new polymer fraction’s recycling burdens [2]. It should be noted that direct comparison of published life cycle assessment (LCA) studies remains challenging due to differences in functional units, system boundaries, allocation procedures, regional electricity mixes, and end-of-life assumptions. Consequently, environmental rankings reported for steel, aluminum, and composite battery housings may vary significantly between studies. Future assessments would benefit from the adoption of harmonized cradle-to-grave methodologies to improve comparability and support more robust sustainability-driven material selection.

Studies on SMCs with functional additives show that fillers, like expanded graphite, form a carbonized surface, reducing the interior pyrolysis rate and enhancing self-extinguishing and electromagnetic shielding [72]. However, these fire-resistant modifications may increase material heterogeneity and impede matrix separation during recycling, which is important for practical DfR strategies and LCA analyses. The recovery of polymer matrices is crucial for recycling and minimizing hard-to-recycle materials. DfR principles highlight that material selection and component release are vital for recycling and reducing hard-to-recycle materials, and adhesive bonds over large areas can improve yield. For polymer matrices, avoiding systems that complicate polymer separation from fibers and metallic elements is essential for feasible matrix recovery [93]. In electrolyte literature, organic and polymeric compounds can be selectively extracted and purified using supercritical CO_2_ and solvents of intermediate polarity. These solvent–CO_2_ hybrid systems recover up to approximately 89% of solvents and conductive salts for reuse in cells. Although this research focuses on liquid electrolytes, it shows that advanced chemical processes can selectively secrete and regenerate complex organic fractions, establishing a framework for future polymer matrix recovery procedures [82,89]. In conclusion, current knowledge indicates that polymer matrix recovery in composite battery housings lies between simple combustion and complete regeneration. Designs enhance the matrix (e.g., rGO) for durability, while efforts are made to develop design tools and chemical processes for selective recovery and reuse, aligning with circular economy principles.

## 8. Circular Design of Enclosures

### 8.1. Integration of Eco-Design Principles

**Figure 3:**
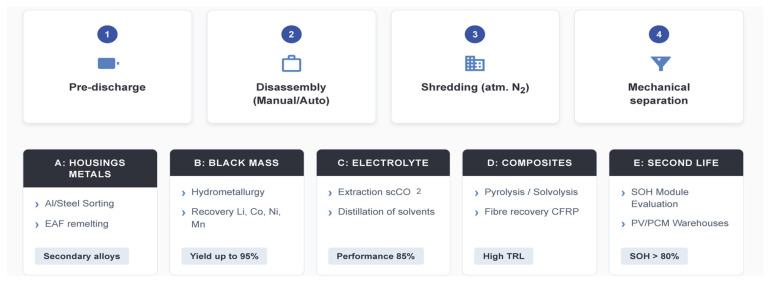
End-of-life package recycling paths [81,82,88,89].

Integrating eco-design principles into battery enclosures requires considering the entire product life cycle, including material selection, design, assembly, and end-of-life scenarios. Design for recycling (DfR) views recycling as a crucial step in circular strategies after remanufacturing, repair, and reuse. Design decisions should prioritize module dismantling and reuse, facilitating material recovery during recycling. DfR requires understanding target recycling processes, as aligning materials and coatings with pathways (e.g., hydrometallurgy, pyrometallurgy) can reduce chemical interactions and enhance recovery. A key eco-design component is design for dismantling (DfD). Reversible connectors and modular architecture promote repair, reuse, and recycling, allowing easy extraction of components like batteries for end-of-life processes [81,93]. DfR guidelines emphasize minimizing inseparable adhesives, especially between incompatible materials, as they hinder material release and reduce value yield. For traction batteries, it is recommended to facilitate package removal, identify cell chemistry and align housing compatibility with recycling processes; rigid polymer housings support recycling [93]. Beyond design, information tools ensure data flow on battery material composition and history. Material passports and “circularity passports” use tracking technology with digital infrastructure and blockchains to gather reliable circular economy data throughout a product’s life cycle. This data informs decisions on reuse, remanufacture, or recycling of components, reducing uncertainties about quality and composition [84]. For traction batteries, regulations like the Chinese standard GB/T 3404-2017 require manufacturers to design easily disassembled packages, incorporating coding and labeling systems for identifying and tracing packages, modules, and cells [80]. Similar initiatives, including the “battery passport” for the European market, aim to ensure transparency in material flows and environmental parameters. Eco-design must address economic and organizational barriers to circular strategies for batteries. Lack of design standards for EVBs, dismantling challenges, and limited technology for diagnostics and tracking cell health increase reuse and recycling costs, reducing circular economy cost-effectiveness. Enablers include end-of-life design for EVBs (DfD), battery modularization, and digitized information and interface standards for data sharing among manufacturers and recyclers [83]. Enclosure design should incorporate modularity, connector accessibility, and tags for sorting. Joining techniques affect dismantling and recovery quality: traditional welding hinders material separation and degrades properties. Additive technologies and DfAM-compliant processes allow complex, modular structures with disassembly points, reducing material loss but requiring manufacturing and cost feasibility assessments [99]. Eco-design in battery housings is vital for the circular economy. Beyond recycling, “internal loops” like repair, reuse, and remanufacturing extend product life and defer raw material demand. Modular, serviceable enclosures with second-life scenarios enhance these loops, provided regulatory frameworks and dismantling infrastructure exist. Life cycle analyses show cascading battery use and advanced V2X interactions delay recycling and reduce the recycled battery share. Second-life scenarios and identification requirements should be design criteria, as usage changes affect emission balance and circular chain efficiency [83,100]. Coordinated policy, technical, and business actions are needed for product design, business models, and recycling technology to reinforce battery system circularity. Figure 3 shows end of life package recycling paths.

### 8.2. Structure Optimization for Recycling

Optimizing enclosure structures for recycling requires aligning material selection with end-of-life processing pathways. The Design for Recycling (DfR) approach involves identifying target material pathways during design and ensuring geometry and composition facilitate material separation. Composite materials challenge current recycling technologies. Using a single aluminum alloy and avoiding copper in package shells simplifies metal recovery. Designs should integrate cells and modules without hindering material separation; excessive bonding complicates economic and disassembly balance. DfR and Design for Disassembly (DfD) principles focus on minimizing material types, using recyclable plastics and avoiding hazardous substances. DfD guidelines promote modular structures, limited variants, minimal materials, no coatings or adhesives, and detachable connectors. Components should be lightweight and easy to handle, addressing panels and profiles often heavily coated. DfD research shows design-to-assembly is not a simple reversal of design-to-dismantling; connectors for rapid installation may be hard to separate, requiring evaluation of connection “separability.” DfR principles highlight material selection and release possibilities. Reducing hard-to-recycle materials and inseparable adhesives, especially on large surfaces, enhances recovery efficiency. For batteries, removing packaging and rigid housing aids recycling, and aligning battery shell chemistry with processes prevents undesirable reactions. Recommendations include clear labeling of battery materials to aid sorting at recycling plants [93]. Structural optimization reduces material complexity and pursues homogeneous scrap streams. DfR analysis for batteries shows designing for separation by industrial processes to the module level is feasible, while single-cell separation is impractical. This requires module and housing geometry for efficient modular unit separation in specialized processing chains, including direct cathode recycling. Consistent aluminum alloy types and fewer metal components in frames and housing mitigate alloy downcycling risks during smelting, preserving secondary metal quality [45]. Structural optimization also involves a labeling system for input stream classification, using stickers, symbols, engravings, barcodes, RFID, blockchain, and material passports; the critical aspect is the label’s information content. Clear labeling helps recyclers classify batches and enhances recycling; standardized labels boost efficiency. The enclosure design should include surfaces for permanent material marking, ensuring information availability even after partial degradation or disassembly during the end-of-life phase. Design for Recycling (DfR) encourages products made from recycled materials, reinforcing production system circularity and fostering a secondary raw materials market, incentivizing future DfR improvement [93]. For battery housings, this involves integrating recycled aluminum components and composites, meeting mechanical and safety requirements.

### 8.3. Modularity of Design

**Figure 4:**
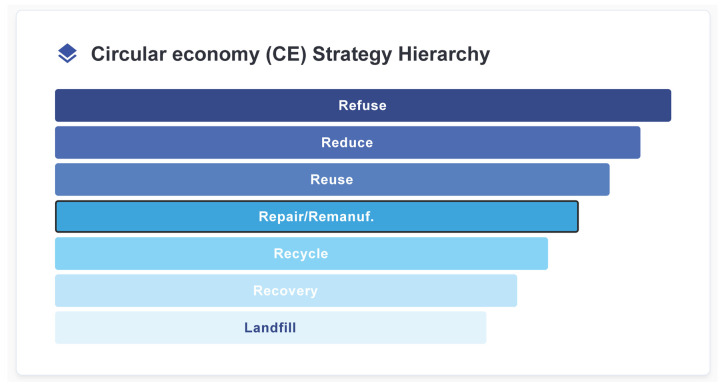
Circular economy (CE) strategy hierarchy [83,84,93].

Modularity in battery systems divides a product into independent units, shaped by design decisions. Kampker notes that defining product architecture and module scope is strategic, based on customer value and supply chain competencies. Effective division enables agile design and early module validation. In battery systems, mechanical, electrical, and thermal modules develop independently but must remain coherent. Conventional frameworks lack modularity for innovations like cell-to-chassis solutions, leading to incompatible outcomes and limiting future system mapping, with no evaluated concepts linking functional and product structures [17,58]. Limited modularity hinders adaptation to circular economy needs. Life cycle engineering design methods shape enclosure modularity. Sakundarini et al. suggest grouping components by end-of-life scenarios to enhance reuse, separating second-life modules from recycling components [101]. Another author advises a permanent product “core” with a replaceable periphery, separating a durable frame from replaceable layers [45]. Modularity is vital for efficient end-of-life processes of lithium-ion batteries. Battery modularization (ET2) cuts handling, transportation, and recovery costs while improving recycling and reuse scalability [83]. Easy module disassembly aids automation, reducing labor and risk, while modular units assist in diagnostics and end-of-life scenarios. This requires standardizing module dimensions and interfaces, affecting package housing geometry and function. Design for disassembly needs modularity with suitable connections. DfD guidelines favor detachable, standard connectors over permanent welds, reducing material variants. For housings, modules should be separable from frames without damaging connections, aiding service and recycling. Design-to-assembly differs from design-to-dismantling; connectors ideal for assembly may hinder separability, requiring modular architecture compromises [45]. In the circular economy, enclosure modularity must align with supply chains and business models. An analysis of CE barriers for traction batteries shows that modular packages with EVB standards and information exchange improve end-of-life processes, aiding recycling and second-life scenarios. Material passports and raw material flow tracking highlight modules with defined functional and material boundaries as information carriers on composition and use history, aiding identification as reusable or recyclable [83,84]. Thus, modularity is central to integrating technical design with circular economy system requirements. Figure 4 shows circular economy strategy hierarchy.

## 9. Future Directions of Development

### 9.1. New Materials with Variable Thermal Conductivity

The concept of materials with spatially controlled thermal conductivity addresses limitations in traditional cooling systems. Active liquid systems require pumps, heat exchangers, and tubing, increasing vehicle weight and chassis complexity [35]. Passive systems with phase-change materials, despite high heat capacity, suffer from low conductivity, increased mass, and volume, and struggle with precise temperature control under variable conditions [49,69,70]. Solutions are needed to enhance conductivity locally without expanding the entire BTMS system. One solution uses composite housing with adjustable conductivity, varying the volume fraction of carbon fibers and conductive materials. High conductor content in “hot spots” creates “heat paths” for passive heat dissipation, maintaining low conductivity where insulation is needed. This could offer a lightweight, maintenance-free thermal management solution, potentially replacing some functions of forced-flow systems. In the CFRP housing study, conductivity anisotropy was considered: k_33_ in thickness is higher than k_11_ and k_22_, aiding directional conduction channels with layer arrangement. A complementary strategy uses local metallic inserts with high conductivity. Copper pins in the composite housing create thermal bridges between the interior and the environment. With copper conductivity at 398 W/mK, the pins act as “spot heat sinks,” connecting interior and outside air with minimal weight gain and negligible impact on stiffness [49]. This approach implements a variable conductivity housing: the CFRP matrix provides high strength and low background conductivity, while the pin network defines conductive heat dissipation paths. Functional coatings are considered an “outer” layer with variable thermal response. One concept uses a passive, climate-adaptive thermochromic layer on the battery housing, modulating optical properties with temperature to reduce solar radiation absorption above 35–45 °C, without sensors or electronics. The proposed solution is cost-effective, scalable, and compatible with existing cell chemistry, enhancing materials with varying conductivity within the housing [51]. Phase change material (PCM) composites are being developed, with conductivity adjusted through high additives. Adding graphene, expanded graphite, boron nitride, or integrating metal foams and porous grates results in highly saturated, cyclically stable structures with enhanced conductivity, suitable for battery cassette housings and upcycling electric vehicle modules in heat storage systems [22,69,70]. In these systems, the housing, typically steel or aluminum, serves as the PCM carrier and thermal conduction interface, with PCM composite properties optimized for balancing latent heat capacity and heat flux [22]. Emerging solutions show materials with variable conductivity can fulfill multiple functions: directional conduction in carbon fiber-reinforced polymer (CFRP) structures with local pins, passive radiation absorption control via thermochromic coatings, and regulated heat distribution in PCM composites with high conductivity fillings [22,49,51]. Integrating these approaches into package enclosures may reduce the weight and complexity of traditional battery thermal management system (BTMS) chips while maintaining essential thermal safety margins. Research on energy storage structures in laminates suggests a thin, large-area design facilitates heat dissipation and may enhance passive conduction path efficiency in variable conductivity housings [102]. However, this multifunctional approach introduces design trade-offs in electrical connectivity, reliability, and operation, which may constrain industrial deployment.

### 9.2. Prospects for the Development of Battery Housings Using Aerogel Layers and Integrated Thermal Runaway Suppression Systems

An analysis of current EV battery housing development suggests future designs should not focus solely on being lightweight. Battery housings are evolving into complex safety systems, providing mechanical protection, electrical insulation, thermal runaway containment, fire resistance, controlled gas discharge, and meeting recycling and dismantling standards. This aligns with the review’s scope, emphasizing the integration of structural, thermal, fire, and circular requirements in one design.

A key development is supporting enclosures and partitions with thin aerogel mats. Silica aerogels and glass fiber-reinforced composites offer low thermal conductivity, low density, and high thermal resistance. Research indicates glass fiber-reinforced SiO_2_ aerogel composites achieve 0.021 W/(m·K) thermal conductivity, high water repellency, and low weight loss at high temperatures, making them suitable barriers in lithium-ion packs [103]. From a housing design perspective, aerogel mats serve multiple functions. They limit heat conduction from thermal runaway cells to neighboring ones, reducing a domino effect risk. They also act as light fire barriers, which is crucial in composite structures where the polymer matrix needs extra high-temperature protection. Aerogel mats can be locally integrated, e.g., between modules or at the housing’s bottom plate, lid, or anticipated hot gas outlet zones, without significantly increasing system weight. This is vital as composites in battery housings aim to reduce weight while maintaining protection.

However, aerogel is not a stand-alone fire safety solution. Its primary role is to delay heat transfer and limit propagation, not to stop thermal runaway in an emergency cell. Thus, designing multilayer housings where aerogel works with cooling systems, gas ventilation, fire protection materials, temperature sensors, and fire suppression solutions is most rational.

The second prospective direction is the use of local reservoirs or capsules with extinguishing liquids, arranged in the case in the form of pockets, inserts, microcapsules or thermally activated layers. The literature describes microencapsulated extinguishing agents containing m.in. Novec 1230, which can release an extinguishing substance in response to an increase in temperature at an early stage of thermal runaway. Such solutions allow you to create self-activating coatings or protective layers around the cells, without the need to start an external fire extinguishing system [104]. Research on microcapsules containing Novec 1230 indicates that these types of additives can reduce the intensity of heat generation and improve the behavior of battery materials in fire conditions. One study showed that the addition of CTS-SA@F7A-Novec 1230 microcapsules to battery composite materials reduced peak heat generation rates and improved cell cooling performance under emergency conditions [105].

From the perspective of enclosure design, the concept of macroscopic “pockets” or local chambers with extinguishing liquid is particularly interesting. They could be deployed in zones that are particularly vulnerable to thermal runaway initiation or propagation, e.g., between modules, under the housing cover, near vent ducts, or in the vicinity of high-voltage components. If a certain temperature is exceeded, the material of the baffle or membrane could be destroyed, releasing the extinguishing agent locally, exactly at the point of danger. This approach may be more material-efficient than globally filling the entire space of the package with extinguishing agent.

However, it is worth noting that Novec 1230 agents, like other pure gaseous agents, suppress flames very well, but may not be enough to completely stop thermal runaway if the package remains strongly heated. In practice, this means that systems based on the Novec 1230 should be treated as an element of the failure reduction system, and not as the only protection. Industry and research literature indicate that gaseous extinguishing agents can reduce flame combustion, but the problem remains to remove heat and prevent re-ignition.

For this reason, hybrid solutions seem to be the most promising, combining:

1. Aerogel mats as a barrier delaying heat transfer;
2. Local capsules or pockets with extinguishing agent;
3. Controlled gas discharge channels;
4. Temperature and pressure sensors;
5. Recycling-compatible fire retardant materials;
6. Modular design for easy disassembly and separation of materials.

This approach addresses one of the main research gaps identified in this review, namely the difficulty of meeting the requirements of fire safety, low weight, durability, cost and recyclability of composite enclosures at the same time.

Future research should focus on developing multilayer enclosures where each layer has a specific function: structural, insulating, fireproof, ventilation, and active damping. From a circular economy perspective, it is crucial that these layers allow dismantling, repair, or recycling, avoiding irreversibly connected, hard-to-separate multi-material systems unless justified by critical safety needs. Battery enclosures should advance beyond replacing materials like aluminum or steel with lighter composites. Designing intelligent, multifunctional safety enclosures with thin aerogel barriers, fire protection layers and locally activated extinguishing agent reservoirs is more promising. These solutions enhance BMS response time, reduce thermal runaway propagation, lower secondary fire risk, and improve safety for users and emergency services. Implementation requires further research on durability, material compatibility, recycling impact, and behavior in modular and package tests.

As shown in Table 8, the suitability of emerging battery housing technologies strongly depends on the battery pack architecture. Aerogel-based thermal barriers and integrated fire suppression systems appear particularly attractive for CTP and CTC configurations due to the reduced spacing between cells and the increased importance of thermal runaway mitigation. In contrast, smart housings and self-sensing composite structures may be implemented across all battery architectures, providing enhanced monitoring and predictive maintenance capabilities. Despite their significant potential, large-scale industrial deployment remains constrained by factors such as manufacturing complexity, material costs, certification requirements, recyclability considerations, and compatibility with existing automotive production systems. Therefore, future development efforts should focus not only on improving performance but also on ensuring scalability and economic viability for mass-market electric vehicles.

### 9.3. Smart Sensor Housing

The integration of sensors and monitoring systems within the package housing is linked with the Battery Management System (BMS) and the thermal management system. The BMS protects the battery from exceeding voltage, current, and temperature limits, while monitoring charge and health through sensors [106,107]. Sensor data is collected by the measurement layer and sent to the control unit, aiding decisions on charging, discharging, and high-voltage disconnection. The enclosure must support sensor sites, beam routing and electromagnetic shielding for reliable monitoring [108]. Temperature, voltage, and current sensors detect hazards like overheating and cell discrepancies. Thermocouples or temperature sensors’ placement affects temperature distribution reliability and safety condition identification. Fiber optic Fiber Bragg Grating (FBG) sensors, resistant to electromagnetic interference, are explored as alternatives for monitoring multiple points. These can be integrated into the housing, providing real-time thermal and mechanical package status. Besides thermal parameters, biochemical parameters, like gases from cell degradation, are monitored. CO_2_ and hydrocarbons measurement differentiates between normal, overload, overdischarge, and internal short-circuit states. An electrolyte leak detection system with a gas sensor can halt charging or discharging upon leak detection [21]. Integrating gas and smoke sensors into the housing creates an “early warning” layer for thermal runaway and facilitates emergency procedures. A comprehensive communication subsystem is essential for using sensor data within the housing for control and diagnostics. The standard BMS architecture uses a Controller Area Network (CAN) bus to exchange information between monitoring systems, the control unit, and external systems [106]. Contemporary solutions use wireless communication technologies like ZigBee, Wi-Fi, GSM, Bluetooth, GPRS, or GPS, enabling remote monitoring and integration with IoT, big data, and cloud computing platforms. This integration underpins “smart” housings, linking sensors to remote diagnostics and algorithm updates. Monitoring systems benefit from cloud analytics and real-time data processing. A solution aligns thermal and biochemical package parameters with the cloud, enabling remote evaluation of module failure, aging, and degradation and providing early warnings. Studies highlight AI and machine learning in automating battery sorting, component identification, and recycling optimization, emphasizing sensor-generated data’s role in end-of-life management. With AIDC and IoT technologies like RFID, NFC, or Bluetooth, enclosures can have digital identities linked to cloud-based status and usage history [80]. Operationally, advanced BMS systems using data processing, modeling, and sensors ensure safe battery operation, extend lifespan, and support vehicle functions, including thermal management and communication. Expanding the enclosure sensor layer, along with wired and wireless communication and digital platform integration, forms the basis of “smart” enclosures, protecting the package and acting as a diagnostic and energy management system component.

## 10. Conclusions

An examination of electric vehicle (EV) battery housing materials and designs reveals that transitioning from steel and aluminum solutions to polymer and carbon fiber composites offers substantial advantages in terms of weight reduction, corrosion resistance, and the integration of structural, thermal, and protective functions. The findings of the study suggest that designing enclosures from a life-cycle perspective necessitates the concurrent consideration of mechanical safety, energy efficiency, and recyclability, aligning with the principles of the circular economy. Composites provide high specific strength and potential for lightweight construction; however, their large-scale implementation poses challenges related to production costs and recycling, particularly for thermosetting dies. The incorporation of eco-design principles, such as design for disassembly, structure optimization for recycling, and modularity, can enhance the efficiency of end-of-life processes and support module reuse scenarios. Mechanical and chemical recycling technologies, including the recovery of metals, fibers, and polymer matrices, require designs to be compatible with the anticipated processing pathways. Future innovations, such as locally controlled thermal conductivity materials, additive manufacturing, and smart sensor housings, can further enhance thermal management, operational safety, and integration with diagnostic systems. The successful implementation of these innovations necessitates coordinated technical, regulatory, and business efforts to ensure that product design, business models, and recycling infrastructure mutually reinforce each other, thereby achieving genuine circularity of battery systems.

## Figures and Tables

**Table 1:** List of abbreviations used in the manuscript.

Abbreviation	Full Term
ABS	Acrylonitrile Butadiene Styrene
AC	Air Conditioning
ABAQUS	Advanced Finite Element Analysis Software
BDU	Battery Disconnect Unit
BEV	Battery Electric Vehicle
BMC	Bulk Molding Compound
BMS	Battery Management System
BTMS	Battery Thermal Management System
CAN	Controller Area Network
CFRP	Carbon Fiber Reinforced Polymer
CMU	Cell Monitoring Unit
CSC	Cell Supervisory Circuit
EAF	Electric Arc Furnace
EMI	Electromagnetic Interference
ERV	Energy Reduction Value
EV	Electric Vehicle
FEM	Finite Element Method
FMVSS	Federal Motor Vehicle Safety Standards
FRP	Fiber-Reinforced Polymer
GFRP	Glass Fiber-Reinforced Polymer
GO	Graphene Oxide
GTR	Global Technical Regulation
HEV	Hybrid Electric Vehicle
HVAC	Heating, Ventilation and Air Conditioning
HV	High Voltage
HVIL	High Voltage Interlock Loop
ISO	International Organization for Standardization
LCA	Life Cycle Assessment
LFP	Lithium Iron Phosphate
LV	Low Voltage
NMC	Lithium Nickel Manganese Cobalt Oxide
PCM	Phase Change Material
PC/PBT	Polycarbonate/Polybutylene Terephthalate Blend
PMU	Pack Monitoring Unit
PPE	Polyphenylene Ether
rGO	Reduced Graphene Oxide
RTM	Resin Transfer Molding
SEA	Specific Energy Absorption
SMC	Sheet Molding Compound
SWOT	Strengths, Weaknesses, Opportunities and Threats
TEC	Thermoelectric Cooling
TIM	Thermal Interface Material
TRL	Technology Readiness Level
UN ECE	United Nations Economic Commission for Europe
VARTM	Vacuum-Assisted Resin Transfer Molding
VW MEB	Volkswagen Modular Electric Drive Matrix
WTW	Well-to-Wheel

**Table 2:** Comparative assessment of numerical simulation and data-driven approaches applied to EV battery enclosure development, including their applications, advantages and limitations [23,33,35,36].

Method	Main Application	Advantages	Limitations
FEM	Crash, stiffness, vibration	High accuracy	High computational cost
CFD	Cooling analysis	Detailed thermal behavior	Long calculation time
Topology optimization	Weight reduction	Material savings	Requires validated FEM
ML/AI	Rapid design screening	Very fast prediction	Requires training datasets
Digital twins	Real-time monitoring	Predictive maintenance	High implementation complexity

**Table 4:** Classification of thermal management systems (BTMSs) [21,29,40,48,49].

**Passive air cooling** Characteristics: Natural convection, no moving parts Cost: Low/Minimum Application: Small Vehicles, Hybrids (HEV), Low Loads	**Active air cooling** Characteristics: Forced flow (fans), integration with HVAC Cost: Low-Medium/Moderate Application: Standard BEV, Older Car Models
**Liquid cooling (indirect)** Characteristics: Cooling plates, water and glycol circulation. Cost: High Application: Most modern BEVs (Tesla, VW MEB)	**Direct refrigerant cooling** Characteristic: AC refrigerant directly included Cost: High Application: Premium Vehicles, Fast Charging
**Phase Change Materials (PCM)** Characteristic: Latent heat storage, passive buffer Cost: Medium Application: Hybrid systems, thermal runaway protection	**Thermoelectric Systems (TEC)** Characteristics: Peltier effect, precise heating and cooling Cost: High/Extra High Application: local cell temperature stabilization, prototypes

**Table 5:** Properties of EV battery housing materials [2,9,16,69,70].

Property	Steel	Aluminum	CFRP	GFRP	Hybrid Composite
Density [g/cm^3^]	7.8	2.7	1.5–2.0	1.8–2.2	2.0–2.5
Specific strength	Low	Medium	Very high	High	High
Weight reduction vs. steel	-	11–25%	Up to 56%	Up to 30–40%	Up to 35%
Corrosion resistance	Low (Coatings)	Good (passive)	Excellent	Excellent	Varied
Recyclability	High	High	Difficult (pyrolyze)	Difficult	Complex
Cost of material	Low	Medium	High	Medium	Variable
TRL in EV housing	High	High	Medium–high	Medium	Low–medium

**Table 6:** Composite enclosure manufacturing technologies.

Technology	Material	Cycle Time	Complexity	Mold Cost	EV Application
RTM/VARTM	CFRP/GFRF	Minutes–hours	High	Medium	Housings, supporting structures
SMC/BMC	Polyester/Vinyl	Short	Large panels	High	Top and bottom covers
Pultrusion	Profile	Continuous	Low	Low	Thresholds, reinforcing beams
Thermoforming	Thermoplastics	Very fast	Limited	Low	Cover elements
3D printing	CF/Al	Long	Extreme	-	Prototypes, BTMS channels

**Table 7:** Comparison of composite recycling methods, technology maturity, industrial feasibility, and circular economy potential for EV battery housing materials [34,82,88,89].

Method	Fiber Yield	Retention of Properties	TRL	Relative Cost	Main Products	Typical Industrial Applications	Economic Attractiveness	Circular Economy Potential
Mechanical recycling	High (short fibers)	Low	9	Low	Fillers, short fibers, polymer granulates	Automotive fillers, underbody components, non-structural composite parts	High	Medium
Pyrolysis	High (long fibers)	High	8	Medium	Recovered carbon fibers	Recycled CFRP for automotive and aerospace components	Medium	High
Solvolysis/Hydrolysis	Very high	Very high	5–6	High	Near-virgin carbon fibers	High-value CFRP recovery and closed-loop recycling applications	Medium	Very High
Combustion (energy recovery)	–	–	9	Low	Heat, electricity	Mixed composite waste streams and municipal waste treatment facilities	Low	Low

**Table 8:** Emerging battery housing technologies, their compatibility with EV battery pack architectures, and implementation challenges based on the reviewed literature [18,21,37,49,75].

Technology	CTM	CTP	CTC	Main Benefits	Key Industrial Challenges
Smart battery housings	High	High	High	Structural health monitoring, predictive maintenance	Sensor integration, data management, additional cost
Aerogel thermal barriers	Medium	High	High	Superior thermal insulation, thermal runaway mitigation	High material cost, manufacturing integration
Fire suppression systems	Medium	High	High	Improved safety and thermal runaway containment	Packaging complexity, maintenance requirements
Structural battery concepts	Low	Medium	High	Weight reduction, increased energy density	Repairability, certification, manufacturing complexity
Self-sensing composite materials	Medium	Medium	High	Real-time damage detection	Industrial maturity, standardization

## Data Availability

No new data were created or analyzed in this study. Data sharing is not applicable to this article.

## References

[1] Yan L., Xu H. (2025). Lightweight Composite Materials in Automotive Engineering: State-of-the-Art and Future Trends. Alex. Eng. J..

[2] Soragaon J. (2025). Development of Lightweight, Sustainable and Multifunctional Battery Pack Materials in Electric Vehicles. Master’s Thesis.

[3] Singh M., Palaniappan S., Rangappa S., Siengchin S. (2025). Emerging Trends in Nanofiller Composites for Aerospace and Automobile Performance Enhancement. J. Eng. Appl. Sci..

[4] Ladpli P., Kopsaftopoulos F., Nardari R., Chang F.-K., Meyendorf N.G. (2017). Battery Charge and Health State Monitoring via Ultrasonic Guided-Wave-Based Methods Using Built-in Piezoelectric Transducers. Smart Materials and Nondestructive Evaluation for Energy Systems 2017.

[5] Phiri R., Mavinkere Rangappa S., Siengchin S., Oladijo O.P., Ozbakkaloglu T. (2024). Advances in Lightweight Composite Structures and Manufacturing Technologies: A Comprehensive Review. Heliyon.

[6] Obrecht M., Singh R., Zorman T. (2022). Conceptualizing a New Circular Economy Feature—Storing Renewable Electricity in Batteries beyond EV End-of-Life: The Case of Slovenia. Int. J. Product. Perform. Manag..

[7] Naresh G., Praveenkumar T., Madheswaran D.K., Solomon J.M., Goud Kureli S., Kolhe Y.K., Lalvani J I.J.R. (2024). Advancing Structural Efficacy and Resonance Performance of Battery Enclosures through Multi-Objective Optimization. J. Low Freq. Noise Vib. Act. Control..

[8] McGinnis R. (2020). CO_2_-to-Fuels Renewable Gasoline and Jet Fuel Can Soon Be Price Competitive with Fossil Fuels. Joule.

[9] Singh A.P., Awasthi S.K. (2025). Analogical Comparison between Functional Safety of Electric Four-Wheelers and Two-Wheelers. Proceedings of the 2025 International Conference on Signal Processing, Computation, Electronics, Power and Telecommunication (IConSCEPT), Karaikal, India, 6 December 2025.

[10] See K.W., Wang G., Zhang Y., Wang Y., Meng L., Gu X., Zhang N., Lim K.C., Zhao L., Xie B. (2022). Critical Review and Functional Safety of a Battery Management System for Large-Scale Lithium-Ion Battery Pack Technologies. Int. J. Coal Sci. Technol..

[11] Czerwinski F. (2021). Current Trends in Automotive Lightweighting Strategies and Materials. Materials.

[12] Hertwich E.G., Ali S., Ciacci L., Fishman T., Heeren N., Masanet E., Asghari F.N., Olivetti E., Pauliuk S., Tu Q. (2019). Material Efficiency Strategies to Reducing Greenhouse Gas Emissions Associated with Buildings, Vehicles, and Electronics—A Review. Environ. Res. Lett..

[13] Jimenez-Martinez M., Valencia-Sánchez J.L., Torres-Cedillo S.G., Cortés-Pérez J. (2024). Battery Housing for Electric Vehicles, a Durability Assessment Review. Designs.

[14] Machín A., Cotto M.C., Díaz F., Duconge J., Morant C., Márquez F. (2024). Environmental Aspects and Recycling of Solid-State Batteries: A Comprehensive Review. Batteries.

[15] Sanguesa J.A., Torres-Sanz V., Garrido P., Martinez F.J., Marquez-Barja J.M. (2021). A Review on Electric Vehicles: Technologies and Challenges. Smart Cities.

[16] Ralls A.M., Leong K., Clayton J., Fuelling P., Mercer C., Navarro V., Menezes P.L. (2023). The Role of Lithium-Ion Batteries in the Growing Trend of Electric Vehicles. Materials.

[17] Achim K., Heimes H.H., Offermanns C., Sasse K., Frieges M.H., Spath B. (2022). Domain Based Product Architecture Approach for Innovative Battery System Design. Proceedings of the 2022 International Symposium on Electromobility (ISEM), Puebla, Mexico, 17 October 2022.

[18] Dhoke A., Dalavi A. (2021). A Critical Review on Lightweight Design of Battery Pack Enclosure for Electric Vehicles. Int. J. Sustain. Transp. Technol..

[19] Krüger C., Spohr S., Merdivan D., Urban P. (2022). Avoiding Structural Redundancies between the Vehicle Body and the Battery Housing Based on a Functional Integration Approach. Automot. Engine Technol..

[20] Gören T. (2024). Concepts for Battery Enclosure. Master’s Thesis.

[21] Wang Y., Gao Q., Wang G., Lu P., Zhao M., Bao W. (2018). A Review on Research Status and Key Technologies of Battery Thermal Management and Its Enhanced Safety. Int. J. Energy Res..

[22] Rajagopal M. (2025). Upcycling End-of-Life EV Batteries into Modular Latent Heat Storage Units for Sustainable District Heating. Next Res..

[23] Zhu J., Wierzbicki T., Li W. (2018). A Review of Safety-Focused Mechanical Modeling of Commercial Lithium-Ion Batteries. J. Power Sources.

[24] Zhu J., Zhang X., Wierzbicki T., Xia Y., Chen G. (2018). Structural Designs for Electric Vehicle Battery Pack against Ground Impact.

[25] Kulkarni S.S., Hale F., Taufique M.F.N., Soulami A., Devanathan R. (2023). Investigation of Crashworthiness of Carbon Fiber-Based Electric Vehicle Battery Enclosure Using Finite Element Analysis. Appl. Compos. Mater..

[26] Hu R., Zhou D., Jia Y., Chen Y., Zhang C. (2024). Dynamic Mechanical Behaviors of Load-Bearing Battery Structure upon Low-Velocity Impact Loading in Electric Vehicles. eTransportation.

[27] Fragiadaki A., Tserpes K. (2025). Numerical Simulation of Dynamic Response of a Composite Battery Housing for Transport Applications. Eng. Proc..

[28] Fragiadaki A., Tserpes K. (2025). Impact Response of a Thermoplastic Battery Housing for Transport Applications. Batteries.

[29] Zhang X., Lin Q., Xiao Y., Jia L., Yang T., Wang L., Ma Q., Wang B. (2025). Top-Down Design Approach of Lightweight Composite Battery Pack Enclosure for Electric Vehicles Based on Numerical Modeling and Topology Optimization. Polymers.

[30] Xiong Y., Pan Y., Wu L., Liu B. (2021). Effective Weight-Reduction- and Crashworthiness-Analysis of a Vehicle’s Battery-Pack System via Orthogonal Experimental Design and Response Surface Methodology. Eng. Fail. Anal..

[31] Shaikh S.A., Taufique M.F.N., Balusu K., Kulkarni S.S., Hale F., Oleson J., Devanathan R., Soulami A. (2024). Finite Element Analysis and Machine Learning Guided Design of Carbon Fiber Organosheet-Based Battery Enclosures for Crashworthiness. Appl. Compos. Mater..

[32] Arslan M., Karamangil M.İ. (2025). Comprehensive Optimization and Design of an Electric Vehicle Battery Box Side Profile for Lightweight and Crashworthiness Using a Novel Hybrid Structure. Appl. Sci..

[33] De Sio P., Gaito M., Esperto V., Cozzolino E., Astarita A., Tucci F. (2025). Life Cycle Assessment of a Composite Prototype Battery Enclosure for Electric Vehicles. Sustainability.

[34] Hermansson F., Edgren F., Xu J., Asp L.E., Janssen M., Svanström M. (2023). Climate Impact and Energy Use of Structural Battery Composites in Electrical Vehicles—A Comparative Prospective Life Cycle Assessment. Int. J. Life Cycle Assess..

[35] Bala A., Kamaraju M.C. (2020). Design and Optimization of Battery Housing in Electric Cars. Master’s Thesis.

[36] Badran M.A., Toha S.F. (2024). Employment of Artificial Intelligence (AI) Techniques in Battery Management System (BMS) for Electric Vehicles (EV): Issues and Challenges. Pertanika J. Sci. Technol..

[37] Deng J., Bae C., Denlinger A., Miller T. (2020). Electric Vehicles Batteries: Requirements and Challenges. Joule.

[38] Belingardi G., Scattina A. (2023). Battery Pack and Underbody: Integration in the Structure Design for Battery Electric Vehicles—Challenges and Solutions. Vehicles.

[39] Upadhye S. (2025). Composite-Based Battery Enclosure (Dissertation). Master’s Thesis.

[40] Singh R., Budarayavalasa S. (2021). Solidification and Stabilization of Hazardous Wastes Using Geopolymers as Sustainable Binders. J. Mater. Cycles Waste Manag..

[41] Rong J.-Q., Rong Y., Liu H., Feng X.-Q., Zhao Z.-L. (2025). Structural Topology Optimization Method with Adaptive Support Design. Adv. Eng. Softw..

[42] Cascino A., Meli E., Rindi A. (2026). Lightweight Design and Topology Optimization of a Railway Motor Support Under Manufacturing and Adaptive Stress Constraints. Vehicles.

[43] Lipman T.E., Maier P. (2021). Advanced Materials Supply Considerations for Electric Vehicle Applications. MRS Bull..

[44] Arora S., Kapoor A., Shen W. (2018). Application of Robust Design Methodology to Battery Packs for Electric Vehicles: Identification of Critical Technical Requirements for Modular Architecture. Batteries.

[45] Maltoni F. (2024). Design of Automotive Battery Systems for the Circular Economy. Ph.D. Thesis.

[46] Azizighalehsari S., Venugopal P., Pratap Singh D., Batista Soeiro T., Rietveld G. (2024). Empowering Electric Vehicles Batteries: A Comprehensive Look at the Application and Challenges of Second-Life Batteries. Batteries.

[47] Carlstedt D., Asp L.E. (2020). Performance Analysis Framework for Structural Battery Composites in Electric Vehicles. Compos. B Eng..

[48] Wang W., Luo Q., Li B., Wei X., Li L., Yang Z. (2013). Recent Progress in Redox Flow Battery Research and Development. Adv. Funct. Mater..

[49] Samarasinghe T. (2024). Design and Development of Composite Enclosure with Variable Thermal Conductivity for Li-Ion Battery Module. Ph.D. Thesis.

[50] Jiang J., Liaw B., Pistoia G. (2018). Behaviour of Lithium-Ion Batteries in Electric Vehicles: Charging Optimization Methods for Lithium-Ion Batteries.

[51] Barack N.L. (2025). Climate-Adaptive Batteries: Passive Thermal Regulation of Lithium-Ion Batteries Using Thermochromic Functional Surface Films. SSRN.

[52] Manzetti S., Mariasiu F. (2015). Electric Vehicle Battery Technologies: From Present State to Future Systems. Renew. Sustain. Energy Rev..

[53] Galos J., Pattarakunnan K., Best A.S., Kyratzis I.L., Wang C., Mouritz A.P. (2021). Energy Storage Structural Composites with Integrated Lithium-Ion Batteries: A Review. Adv. Mater. Technol..

[54] Hussein H.M., Ibrahim A.M., Taha R.A., Rafin S.M.S.H., Abdelrahman M.S., Kharchouf I., Mohammed O.A. (2024). State-of-the-Art Electric Vehicle Modeling: Architectures, Control, and Regulations. Electronics.

[55] Muratori M., Alexander M., Arent D., Bazilian M., Cazzola P., Dede E.M., Farrell J., Gearhart C., Greene D., Jenn A. (2021). The Rise of Electric Vehicles—2020 Status and Future Expectations. Prog. Energy.

[56] Wüstenhagen S., Beckert P., Lange O., Franze A. (2021). Light Electric Vehicles for Muscle–Battery Electric Mobility in Circular Economy: A Comprehensive Study. Sustainability.

[57] Hesse H., Schimpe M., Kucevic D., Jossen A. (2017). Lithium-Ion Battery Storage for the Grid—A Review of Stationary Battery Storage System Design Tailored for Applications in Modern Power Grids. Energies.

[58] Plocher L., Heller M., Ingendoh B., Turhan H., Burgard M., Wennemar S., Kampker A. (2023). Mini-Environments In Lithium-Ion Battery Cell Production: A Survey On Current State, Challenges And Trends. Proceedings of the Conference on Production Systems and Logistics CPSL 2023-2.

[59] Esen D.Ö., Shabaneh S.A.Y.A. (2026). Integrated Thermal Management Strategies for Electric Vehicles: A System-Level Review of Battery Cooling, Heat Pump Technologies, PCM Integration, and Cold-Climate Performance. J. Therm. Anal. Calorim..

[60] Alawi A., Saeed A., Sharqawy M.H., Al Janaideh M. (2025). A Comprehensive Review of Thermal Management Challenges and Safety Considerations in Lithium-Ion Batteries for Electric Vehicles. Batteries.

[61] Recoskie S., MacNeil D.D., Darcovich K., Perron J., Pedroso S. (2025). Low-Temperature Performance and Durability of Electric Vehicle Battery Cells Under Isothermal Conditions. Energies.

[62] Akinci A., Yilmaz S., Sen U. (2012). Wear Behavior of Basalt Filled Low Density Polyethylene Composites. Appl. Compos. Mater..

[63] Calborean A., Máthé L., Bruj O. (2025). Phase Change Materials for Thermal Management in Lithium-Ion Battery Packs: A Review. Batteries.

[64] Lučić M., Lukić J., Grujić I. (2024). Statistical Analysis of Trends in Battery Electric Vehicles: Special Reference to Vehicle Weight Reduction, Electric Motor, Battery, and Interior Space Dimensions. Arch. Automot. Eng.–Arch. Motoryz..

[65] Ganguli T., Date P.P. (2024). Lightweight Battery Enclosure Design. Proceedings of the 20th Metal Forming International Conference, Krakow, Poland, 15 September 2024.

[66] Gupta P., Toksha B., Patel B., Rushiya Y., Das P., Rahaman M. (2022). Recent Developments and Research Avenues for Polymers in Electric Vehicles. Chem. Rec..

[67] Sripad S., Bills A., Viswanathan V. (2021). A Review of Safety Considerations for Batteries in Aircraft with Electric Propulsion. MRS Bull..

[68] Martínez-Sánchez R., Molina-García A., Ramallo-González A.P. (2024). Regeneration of Hybrid and Electric Vehicle Batteries: State-of-the-Art Review, Current Challenges, and Future Perspectives. Batteries.

[69] Azzopardi B., Hapid A., Kaleg S., Sudirja S., Onggo D., Budiman A.C. (2023). Recent Advances in Battery Pack Polymer Composites. Energies.

[70] Budiman A.C., Azzopardi B., Sudirja, Perdana M.A.P., Kaleg S., Hadiastuti F.S., Hasyim B.A., Amin, Ristiana R., Muharam A. (2023). Phase Change Material Composite Battery Module for Thermal Protection of Electric Vehicles: An Experimental Observation. Energies.

[71] Aguilar Esteva L.C., Kasliwal A., Kinzler M.S., Kim H.C., Keoleian G.A. (2021). Circular Economy Framework for Automobiles: Closing Energy and Material Loops. J. Ind. Ecol..

[72] Höhne C.-C., Blaess P., Ilinzeer S., Griesbaum P. (2023). New Approach for Electric Vehicle Composite Battery Housings: Electromagnetic Shielding and Flame Retardancy of PUR/UP-Based Sheet Moulding Compound. Compos. Part A Appl. Sci. Manuf..

[73] Nie B., Lim J., Liu T., Kovalenko I., Guo K., Liang J., Zhu J., Sun H. (2023). Multifunctional Composite Designs for Structural Energy Storage. Battery Energy.

[74] Ciez R.E., Steingart D. (2020). Asymptotic Cost Analysis of Intercalation Lithium-Ion Systems for Multi-Hour Duration Energy Storage. Joule.

[75] Mohanty A.K., Vivekanandhan S., Tripathi N., Roy P., Snowdon M.R., Drzal L.T., Misra M. (2023). Sustainable Composites for Lightweight and Flame Retardant Parts for Electric Vehicles to Boost Climate Benefits: A Perspective. Compos. Part C Open Access.

[76] Burd J.T.J., Moore E.A., Ezzat H., Kirchain R., Roth R. (2021). Improvements in Electric Vehicle Battery Technology Influence Vehicle Lightweighting and Material Substitution Decisions. Appl. Energy.

[77] Chen J., Cheng F. (2009). Combination of Lightweight Elements and Nanostructured Materials for Batteries. Acc. Chem. Res..

[78] Naik N., Suresh P., Yadav S., Nisha M.P., Arias-Gonzáles J.L., Cotrina-Aliaga J.C., Bhat R., Jalageri M.D., Kaushik Y., Kunjibettu A.B. (2023). A Review on Composite Materials for Energy Harvesting in Electric Vehicles. Energies.

[79] Harper G., Sommerville R., Kendrick E., Driscoll L., Slater P., Stolkin R., Walton A., Christensen P., Heidrich O., Lambert S. (2019). Recycling Lithium-Ion Batteries from Electric Vehicles. Nature.

[80] Pesaran A., Roman L., Kincaide J. (2023). Electric Vehicle Lithium-Ion Battery Life Cycle Management.

[81] Hagelüken C., Goldmann D. (2022). Recycling and Circular Economy—Towards a Closed Loop for Metals in Emerging Clean Technologies. Miner. Econ..

[82] Harper G.D.J., Kendrick E., Anderson P.A., Mrozik W., Christensen P., Lambert S., Greenwood D., Das P.K., Ahmeid M., Milojevic Z. (2023). Roadmap for a Sustainable Circular Economy in Lithium-Ion and Future Battery Technologies. J. Phys. Energy.

[83] Sopha B.M., Purnamasari D.M., Ma’mun S. (2022). Barriers and Enablers of Circular Economy Implementation for Electric-Vehicle Batteries: From Systematic Literature Review to Conceptual Framework. Sustainability.

[84] Babbitt C.W., Althaf S., Cruz Rios F., Bilec M.M., Graedel T.E. (2021). The Role of Design in Circular Economy Solutions for Critical Materials. One Earth.

[85] Rehman S., Al-Greer M., Burn A.S., Short M., Cui X. (2025). High-Volume Battery Recycling: Technical Review of Challenges and Future Directions. Batteries.

[86] Thakur J., Martins Leite de Almeida C., Baskar A.G. (2022). Electric Vehicle Batteries for a Circular Economy: Second Life Batteries as Residential Stationary Storage. J. Clean. Prod..

[87] Elwert T., Goldmann D., Römer F., Buchert M., Merz C., Schueler D., Sutter J. (2015). Current Developments and Challenges in the Recycling of Key Components of (Hybrid) Electric Vehicles. Recycling.

[88] Neumann J., Petranikova M., Meeus M., Gamarra J.D., Younesi R., Winter M., Nowak S. (2022). Recycling of Lithium-Ion Batteries—Current State of the Art, Circular Economy, and Next Generation Recycling. Adv. Energy Mater..

[89] Fan E., Li L., Wang Z., Lin J., Huang Y., Yao Y., Chen R., Wu F. (2020). Sustainable Recycling Technology for Li-Ion Batteries and Beyond: Challenges and Future Prospects. Chem. Rev..

[90] Dörfler S., Althues H., Härtel P., Abendroth T., Schumm B., Kaskel S. (2020). Challenges and Key Parameters of Lithium-Sulfur Batteries on Pouch Cell Level. Joule.

[91] Adewale L.D. (2025). Lifecycle Assessment and Circular Economy Strategies for Sustainable Automotive Materials: Optimizing Recycling, Waste Reduction, and Cost Efficiency. Int. J. Res. Publ. Rev..

[92] Dunn J.B., Gaines L., Kelly J.C., James C., Gallagher K.G. (2015). The Significance of Li-Ion Batteries in Electric Vehicle Life-Cycle Energy and Emissions and Recycling’s Role in Its Reduction. Energy Environ. Sci..

[93] Norgren A., Carpenter A., Heath G. (2020). Design for Recycling Principles Applicable to Selected Clean Energy Technologies: Crystalline-Silicon Photovoltaic Modules, Electric Vehicle Batteries, and Wind Turbine Blades. J. Sustain. Metall..

[94] Charlie N., Daniel O. (2024). Design a High-Voltage Battery Enclosure System Using a New Manufacturing Method Designing a Battery Enclosure Component by Hot Form Quenching Using High Strength Aluminium. Master’s Thesis.

[95] Balasubramani N., El Mansori M. (2026). Impacts of Vehicle Electrification on Al-Si Castings: An Overview of Current Manufacturing Processes, Mechanical Properties, and Applications. Adv. Manuf..

[96] Kalhor A., Dykas J., Rodak K., Grajcar A. (2024). Materials and Constructional Design for Electric Vehicles: A Review. Adv. Sci. Technol. Res. J..

[97] Cusenza M.A., Guarino F., Longo S., Ferraro M., Cellura M. (2019). Energy and Environmental Benefits of Circular Economy Strategies: The Case Study of Reusing Used Batteries from Electric Vehicles. J. Energy Storage.

[98] Hemanth R., Sekar M., Suresha B. (2014). Effects of Fibers and Fillers on Mechanical Properties of Thermoplastic Composites. Indian J. Adv. Chem. Sci..

[99] Yeong W.Y., Sing S.L., Aman B. (2021). Feasibility Study on Topological Optimisation and Additive Manufacturing of an Electric Vehicle Battery Housing. Res. Sq..

[100] Song A., Dan Z., Zheng S., Zhou Y. (2024). An Electricity-Driven Mobility Circular Economy with Lifecycle Carbon Footprints for Climate-Adaptive Carbon Neutrality Transformation. Nat. Commun..

[101] Sakundarini N., Taha Z., Abdul-Rashid S.H., Ghazila R.A.R. (2013). Optimal Multi-Material Selection for Lightweight Design of Automotive Body Assembly Incorporating Recyclability. Mater. Des..

[102] Adam T., Liao G., Petersen J., Geier S., Finke B., Wierach P., Kwade A., Wiedemann M. (2018). Multifunctional Composites for Future Energy Storage in Aerospace Structures. Energies.

[103] Huang Y., Fan Y., Sun L., Shen X., Zhao Y., Cao Y., Wang J., Wang Z. (2025). Mechanism of Heat Transfer Suppression and Safety Evaluation of High-Performance Aerogel Insulation Materials in the Thermal Runaway Propagation of Lithium-Ion Batteries. SSRN.

[104] Zhang W., Wu L., Du J., Tian J., Li Y., Zhao Y., Wu H., Zhong Y., Cao Y.-C., Cheng S. (2021). Fabrication of a Microcapsule Extinguishing Agent with a Core–Shell Structure for Lithium-Ion Battery Fire Safety. Mater. Adv..

[105] Zhou G., Li Y., Liu Y., Zhang Q., Wei Z., Li S., Yang S., Yuan S., Fan T., Huang Q. (2024). Preparation of a Novel Environmental-Friendly Lithium-Ion Battery Fire Suppression Microcapsule and Its Fire Extinguishing Mechanism in Coordination with ABC Dry Powder. J. Clean. Prod..

[106] Khawaja Y., Shankar N., Qiqieh I., Alzubi J., Alzubi O., Nallakaruppan M.K., Padmanaban S. (2023). Battery Management Solutions for Li-Ion Batteries Based on Artificial Intelligence. Ain Shams Eng. J..

[107] Lipu M.S.H., Al Mamun A., Ansari S., Miah M.S., Hasan K., Meraj S.T., Abdolrasol M.G.M., Rahman T., Maruf M.H., Sarker M.R. (2022). Battery Management, Key Technologies, Methods, Issues, and Future Trends of Electric Vehicles: A Pathway toward Achieving Sustainable Development Goals. Batteries.

[108] Collijn M., Johansson E. (2019). Design for Assembly and Disassembly of Battery Packs. Master’s Thesis.

